# Plasma proteome of Long-COVID patients indicates HIF-mediated vasculo-proliferative disease with impact on brain and heart function

**DOI:** 10.1186/s12967-023-04149-9

**Published:** 2023-06-10

**Authors:** Cristiana Iosef, Michael J. Knauer, Michael Nicholson, Logan R. Van Nynatten, Gediminas Cepinskas, Sorin Draghici, Victor K. M. Han, Douglas D. Fraser

**Affiliations:** 1 Children’s Health Research Institute, Victoria Research Laboratories, 800 Commissioners Road East, London, ON N6C 2V5 Canada; 2 Department of Pathology and Laboratory Medicine, London, ON N6A 5C1 Canada; 3 Department of Medicine, Western University, London, ON N6A 5C1 Canada; 4 Lawson Health Research Institute, London, ON N6C 2R5 Canada; 5 Department of Medical Biophysics, Western University, London, ON N6A 5C1 Canada; 6 Department of Computer Science College of Engineering, Wayne State University, Ann Arbor, MI 48202 USA; 7 Department of Pediatrics, Western University, London, ON N6A 5C1 Canada; 8 Department of Physiology & Pharmacology, Western University, London, ON N6A 5C1 Canada; 9 Department of Clinical Neurological Sciences, Western University, London, ON N6A 5C1 Canada; 10 Advaita Bioinformatics, Ann Arbor, 48105-2552 USA; 11 National Science Foundation, Alexandria, VA 22314 USA

## Abstract

**Aims:**

Long-COVID occurs after SARS-CoV-2 infection and results in diverse, prolonged symptoms. The present study aimed to unveil potential mechanisms, and to inform prognosis and treatment.

**Methods:**

Plasma proteome from Long-COVID outpatients was analyzed in comparison to matched acutely ill COVID-19 (mild and severe) inpatients and healthy control subjects. The expression of 3072 protein biomarkers was determined with proximity extension assays and then deconvoluted with multiple bioinformatics tools into both cell types and signaling mechanisms, as well as organ specificity.

**Results:**

Compared to age- and sex-matched acutely ill COVID-19 inpatients and healthy control subjects, Long-COVID outpatients showed natural killer cell redistribution with a dominant resting phenotype, as opposed to active, and neutrophils that formed extracellular traps. This potential resetting of cell phenotypes was reflected in prospective vascular events mediated by both angiopoietin-1 (ANGPT1) and vascular-endothelial growth factor-A (VEGFA). Several markers (ANGPT1, VEGFA, CCR7, CD56, citrullinated histone 3, elastase) were validated by serological methods in additional patient cohorts. Signaling of transforming growth factor-β1 with probable connections to elevated EP/p300 suggested vascular inflammation and tumor necrosis factor-α driven pathways. In addition, a vascular proliferative state associated with hypoxia inducible factor 1 pathway suggested progression from acute COVID-19 to Long-COVID. The vasculo-proliferative process predicted in Long-COVID might contribute to changes in the organ-specific proteome reflective of neurologic and cardiometabolic dysfunction.

**Conclusions:**

Taken together, our findings point to a vasculo-proliferative process in Long-COVID that is likely initiated either prior hypoxia (localized or systemic) and/or stimulatory factors (i.e., cytokines, chemokines, growth factors, angiotensin, etc). Analyses of the plasma proteome, used as a surrogate for cellular signaling, unveiled potential organ-specific prognostic biomarkers and therapeutic targets.

**Supplementary Information:**

The online version contains supplementary material available at 10.1186/s12967-023-04149-9.

## Introduction

The “Long-COVID” syndrome is also referred to as long-haul COVID, post-COVID-19 condition, chronic COVID and post-acute sequelae of SARS-CoV-2 (PASC) [1–3]. Symptoms of Long-COVID can be either similar or dissimilar from those of acute COVID-19. While some patients have symptoms that last for weeks, months or years after the initial diagnosis [2, 3], some recover from COVID-19 and then endure a return of the symptoms, or they acquire new symptoms [4–7]. Additionally, individuals who were asymptomatic during their acute infection can still develop symptoms at a later date [1, 2]. Thus, a spectrum of COVID-19 severities drive Long-COVID symptoms [1–3].

Long-COVID investigations are hampered by the variety of symptoms reported, as well as other contributing factors such as (i) biological sex, (ii) older age, (iii) severity of initial COVID-19 illness, (iv) amplitude of the immune response to initial infection, (v) vaccination status, (vi) the SARS-CoV-2 variant that caused the initial infection, (vii) the severity of any preexisting health conditions (diabetes, lung problems, autoimmune diseases, or obesity), and/or (viii) health care inequities [1, 2, 4–7]. A number of mechanisms have been proposed for Long-COVID, including (i) reactivated SARS-CoV-2 particles [8, 9], (ii) epigenetically programmed, overactive immune cells that chronically release inflammatory substances [1], (iii) autoimmune disease triggered by SARS-CoV-2 infection (10–13), or (iv) a combination of the above factors originating from the initial COVID-19 disease [5, 10–19]. We recently reported that vascular transformation biomarkers were significantly elevated in plasma from Long-COVID outpatients (e.g., ANGPT1, MMP1, VEGF-A, etc.), suggesting that angiogenesis may be a common mechanism in these patients with prolonged and diffuse symptoms [4, 5].

In this study, we analyzed the plasma proteome of Long-COVID outpatients, as a surrogate of cellular/tissue activities, and compared with age- and sex-matched acutely ill COVID-19 inpatients and healthy control subjects. The plasma proteome was deconvoluted into cell-type profiles and signaling pathways, as well as specific organ systems based on previously curated biomarkers [20–30]. Multiple biomarkers were elevated in Long-COVID outpatients and these could point to an immune triggered vascular proliferation secondary to COVID-19 associated hypoxia (local or systemic) or other stimulatory factors, thereby altering brain and heart function [31–34].

## Materials and methods

### Clinical assessment strategies

This study was approved by Western University, Human Research Ethics Board (Long-COVID REB ID# 120084; COVID-19 REB ID# 1670; Healthy Control Subjects REB ID# 6963). All patients were screened and enrolled from our tertiary care hospitals (London Health Sciences Centre, London, Ontario, Canada). Both Long-COVID outpatients and acutely ill COVID-19 inpatients had their COVID-19 status confirmed by standard hospital testing for SARS-CoV-2 viral genes using polymerase chain reaction (PCR). Long-COVID outpatients were referred to a specialty clinic based on prolonged, diffuse symptoms. Venous blood work was drawn once as part of a larger clinical screen, and subsequent analysis was performed on excess plasma collected for routine blood work by Pathology and Laboratory Medicine (PaLM). COVID-19 inpatients were enrolled on hospital admission, either to the medical ward or to the intensive care unit (ICU). Blood from COVID-19 inpatients was obtained from indwelling catheters or a venipuncture as required. The healthy control subjects were individuals without disease, acute illness or prescription medications and were previously banked in the Translational Research Centre, London, ON (Directed by Dr. D.D. Fraser; https://translationalresearchcentre.com/). These latter samples were obtained prior to the emergence of SARS-CoV-2 in our region and therefore, were considered not to have been exposed to the virus. Final participant groups were matched by age and gender (Long-COVID outpatients with acutely ill COVID-19 inpatients and healthy control subjects) [4]. Blood was centrifuged and plasma was isolated and aliquoted in 250 µL, and frozen at − 80 °C until analysis.

### Targeted proteomics

Proximity Extension Assay (PEA) was used to measure plasma protein expression and included immune-recognition with dNTP-labeled antibodies, extension mediated by polymerases, amplification, and detection [30, 35–40]. All clinical samples were analyzed on the same 88 well plate. A control was used to estimate precision, a negative control was used to set background levels and to calculate limit of detection, a plate control to correct levels between plates, and a reference plasma control was used to estimate CV between runs. The relative protein quantification is presented as a Normalized Protein Expression (NPX) on a log2 scale. Data generation of NPX consists of normalization to the extension control, log2-transformation, and level adjustment using the plate control. PEA was outsourced to OLINK laboratories (Boston, MA) [39, 41, 42].

### Bioinformatic analyses

Normalized Protein eXpression (NPX) data was processed to determine the following: (i) differentially expressed biomarkers, (ii) *Gene Ontology* (GO) and *pathways enrichment*, and (ii) affiliation with different *cell types*. Selected biomarkers were investigated for *candidate drugs*. The NPX of each peptide was normalized within the specified protein. All normalization computations used the medians to multiply and/or normalize the data. Multiple Mann Whitney Test analysis was performed to determine the differentially expressed proteins. For the *functional annotation* analysis, data was trained in three steps to be certain that multiple platforms identify similar patterns: (1) analysis with DAVID Bioinformatics Resources (version 6.8; https://david.ncifcrf.gov/) and PANTHER Classification System (version 14.0; http://www.pantherdb.org/), while the protein–protein interaction network analysis was carried out with STRING (Search Tool for the Retrieval of Interacting Genes/Proteins) (version 11.0; https://string-db.org/) [43]; next (2) analysis with Metascape software, a gene annotation and analysis resource (https://metascape.org/gp/index.html#/main/step1) and GSEA, Gene Set Enrichment Analysis platform (https://www.gsea-msigdb.org/gsea/index.jsp); (3) selected biomarkers were analyzed with Kyoto Encyclopedia of Genes and Genomes (KEGG) Mapper software (https://www.genome.jp/kegg/mapper/). Results that converged to the same findings were validated using (i) iPathwayGuide (iPG) (https://www.advaitabio.com) a software program based on KEGG charts (an updated version of KEGG Mapper [44–48] and (ii) Qlucore (https://qlucore.com/geneexpressions). Using iPG, the analysis included the selection of differentially expressed proteins (DEPs) based on a fold change greater than 0.6 (in log2 scale) as well as a p-value lower than 0.05 after the correction for multiple experiments. The pathways were analyzed with the impact analysis approach [44], which takes into consideration the position of each gene on each pathway, as well as the type and direction of all signals throughout the pathway [44]. Adjusted P values less than 0.05 were considered significant for both GO and pathway analysis.

*Immune-cell deconvolution*—was completed by CIBERSORT analysis after mapping proteins onto genes (https://cibersort.stanford.edu). CIBERSORT is an analytical free tool provided by Stanford University (“Stanford”) [49].

*Matrix visualization* was performed by Morpheus software and interacting tools, from Broad Institute (https://software.broadinstitute.org/morpheus). Hierarchical clustering was performed by row, using Pierson one correlation algorithms both for the regular maps and similarity matrixes. K-means analysis was applied for columns to analyze the delineation patterns of the study groups.

*Principal component analysis* was performed with ClustVis software: https://biit.cs.ut.ee/clustvis/.

### Other statistical analyses

Bar graphs summarize data analyzed using Mann–Whitney U tests for unpaired data (two-sided). *P < 0.05; ***P < 0.0001 (GraphPad Prism, version 9). In the analysis process, several public R-studio packages from Bioconductor, were used for the initial global data analysis, as follows: data load (DBI, odbc); data manipulation (tidyr); data visualization (ggplot2, rgl, leaflet); data modeling (tidymodels); data report (shiny); spatial data (maps); web work (xml, github); R writing (devtools, curl).

### Marker validation

ANGPT1 and VEGFA were measured in human plasma using a custom multiplexed immunoassay kit according to manufacturer’s instructions (Endothelial Injury Marker 12-Plex Human ProcartaPlex^™^ Panel, EPX120-15849–901). Other markers were measured by Enzyme-linked Immunosorbent Assay (ELISA). For NK cell marker validation, we used: (a) Human NCAM1 ELISA Kit (CD56) (Abcam: ab119587) and Human CCR7 (Sandwich ELISA) ELISA Kit—(LSBio: LS-F4886). For Neutrophil Extracellular Trap Formation validation, we used EpiQuik Histone H3 Citrullination ELISA Kit (Colorimetric; EpigenTek: P-3095-96) and Human Neutrophil Elastase ELISA Kit (Abcam: ab270204). ELISAs was performed as per the manufacture’s protocols, and plasma was diluted 1:20. For validation, we used random patients from a different cohort than those tested by the targeted proteomics (N = 11). Plasma samples were tested in sets of three technical replicates.

## Results

### Study model and dimensionality reduction of the data sets

**Fig. 1:**
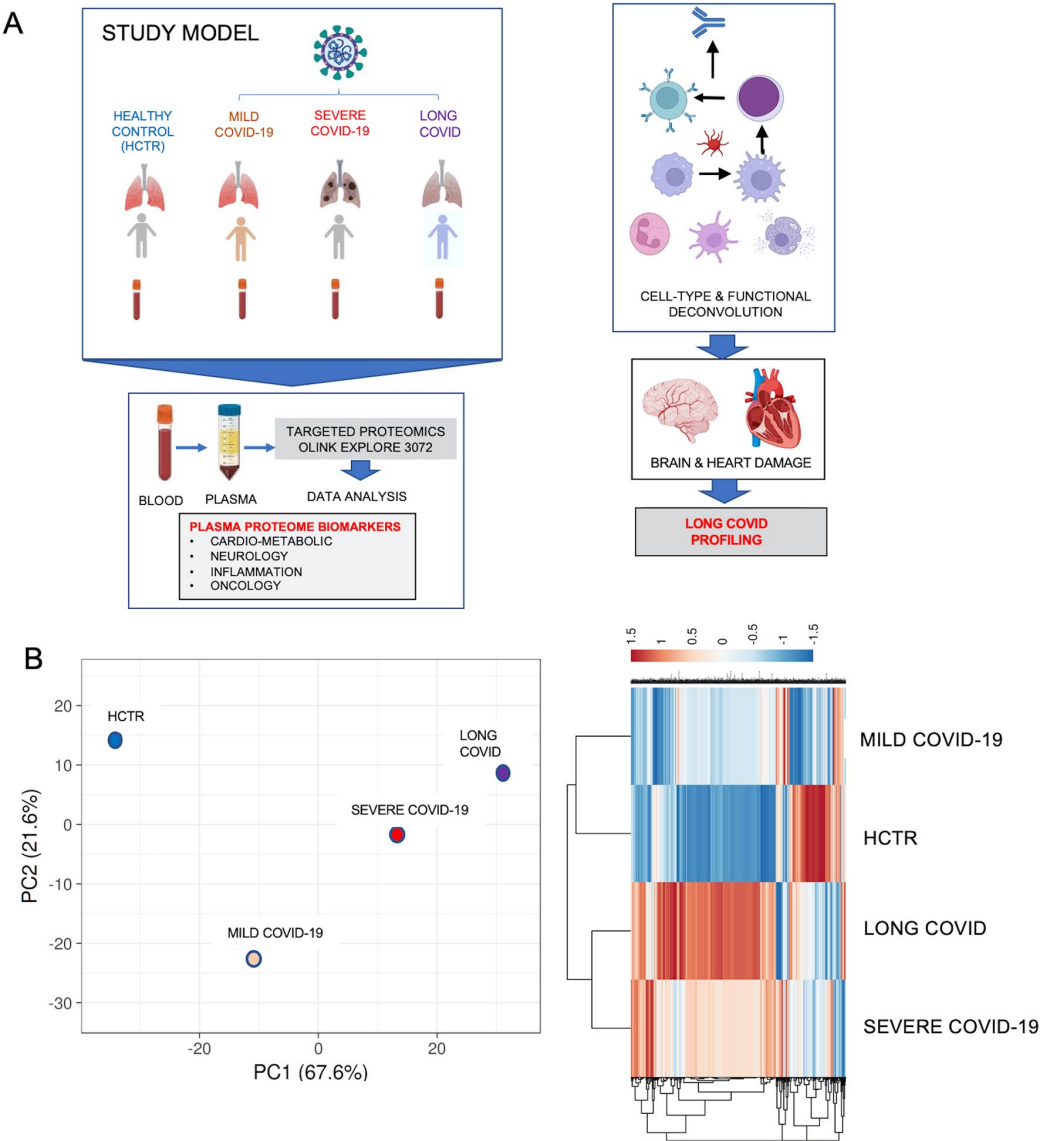
Study model and dimensionality reduction of data sets. **A** Model in the left panel illustrates four study cohorts, including Long-COVID, severe COVID-19, mild COVID-19, and healthy control subjects. The plasma proteome obtained by Proximity Extension Assay (PEA), with the expression of 3072 plasma proteins measured. Right panel informs on the type of data processing strategy targeting cell and organ typing. **B** Principal component analysis and hierarchical clustering. Unit variance scaling was applied to rows; SVD with imputation is used to calculate principal components. X and Y axis show principal component 1 and principal component 2 which explain PC1 (67.6%) and PC2 (21.6%) of the total variance, respectively (N = 4 data points, the means of the values given by all patients within a group). Data was processed by Clustvis software

The patient demographic and clinical data are shown in Additional file 1: Table S1 (Long-COVID outpatients) and Additional file 2: Table S2 (COVID-19 inpatients). Figure 1A (left panel) illustrates the experimental model consisting of four cohorts of patients, Long-COVID outpatients, acutely ill COVID-19 inpatients (both mild and severe) and healthy control subjects. Figure 1A (right panel) shows the data processing strategy. Dimensionality reduction analysis including all data sets, with complete hierarchical clustering, is shown in Additional file 3: Fig S1, where all data points that contributed to the PCA are shown grouped by disease severity; unit variance scaling was applied to rows, and SVD with imputation is used to calculate principal components. In Additional file 3: Fig S1, X and Y axes show principal component 1 and principal component 2 that explain 27.5% and 15.1% of the total variance, respectively (N = 88 data points). To heighten the differences between groups, in Fig. 1B we present the principal components analysis (PCA) and hierarchical clustering of averaged data points. The X and Y axes show principal component 1 and principal component 2, which explain 67.6% and 21.6% of the total variance, respectively. Data was processed by ClustVis software for both Additional file 3: Fig S1 and Fig. 1B. Overall, the data suggested that Long-COVID is a distinct disease compared to COVID-19 ICU inpatients (severe disease), although the cohorts may share some mechanisms. In contrast, the COVID-19 ward inpatients (mild disease) may share mechanisms closer to the healthy control subjects.

### Natural killer (NK) cells of Long-COVID outpatients changed phenotype from activated to resting

**Fig. 2:**
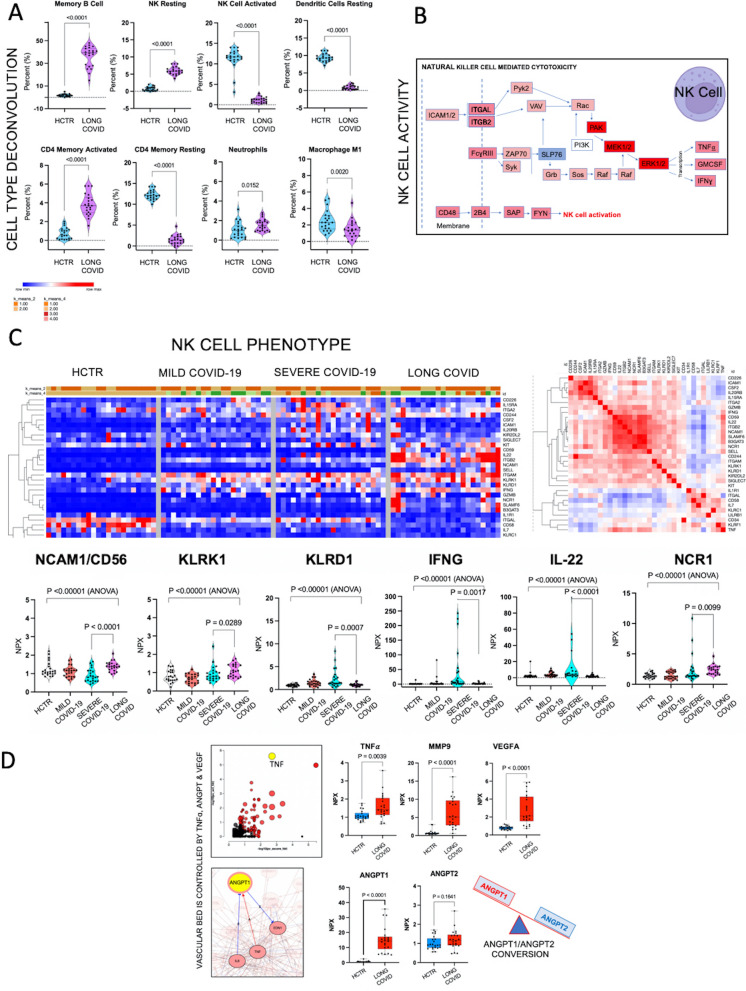
Natural killer (NK) cells change phenotypes in Long-COVID from activated to resting. **A** General immune cell typing using CIBERSORT analysis tool. The algorithms were applied to plasma proteome data sets after proteins were converted into genes. Graphs show the proportional contribution of each cell type in plasma for Long-COVID outpatients compared to healthy control subjects (HCTR). **B** Diagram depicts the NK cell cytotoxic pathways, a segment of KEGG pathways through iPathways Guide platform. Up-regulated biomarkers are shown in red and down-regulated in blue. Red arrows indicate  direct interactions. Analysis was done with KEGG Mapper, also confirmed by iPathwayGuide. **C** Heatmaps represent the profiling of the NK cell phenotype. Left heatmap shows the hierarchical clustering of NK cell hand curated markers and in the right panel the similarity test based on Pearson Correlation. For data visualization we used Morpheus software from Broad Institute. Graphs emphasize  individual NK cell markers expression in plasma compared among study groups. Statistical significance was processed with GraphPad 9, P-value was considered significant if < 0.05 in either ANOVA (group), or Mann–Whitney U test. **D** Left graph represents pathways analysis scoring done with iPathwaysGuide software; tumor necrosis factor (TNF) signaling was the highest hit; schematic below represents the main predicted molecular interactions of ANGPT1 according to STRING analysis and confirmed by iPathwayGuide. Graphs shows the TNF expression in plasma as detected by PEA. The rest of the graphs indicate the plasma expression levels of Angiopoietin 1 (ANGPT1), Matrix metalloproteinase 9 (MMP9) and Vasculo- Endothelial Growth Factor A (VEGFA); all these proteins are predicted to be induced and potentiated by TNF. Statistical significance was processed with GraphPad 9, P-value was considered significant if < 0.05 with Mann–Whitney U test

The plasma proteome was deconvoluted to various immune cell-types from tissues based on their complex OMICS profile using CIBERSORT. The proportion of each cell-type contribution to Long-COVID plasma proteome was compared to healthy control subjects (Fig. 2A). Plasma protein patterns suggest that Long-COVID was associated with a shift in NK cells from activated to resting phenotype. The NK phenotype shift was validated by ELISA; CD56 and CCR7 markers were investigated in cohorts containing 11 patients or subjects, which were different from those included in the targeted proteomic groups. While CD56 is a common marker for all NK cells, CCR7 marks the active phenotype and CCR7 was depressed in the Long-COVID outpatients compared to those with acute disease (Additional file 5: Fig S4A). The NK cell cytotoxic pathways (iPathwaysGuide software) are shown in Fig. 2B. Elevated biomarkers are illustrated in red and depressed biomarkers shown in blue. The original NK-cell signaling KEGG map is presented in Additional file 4: Fig S2. Down-regulation of SLP76, an immune response adaptor, was observed while individual markers such as PAK1, MEK1/2 and ERK1/2 were upregulated and interconnected in signaling cascades that target the secretion of TNFα, IFNγ and GM-CSF. Furthermore, the VAV1 exchange factor for Rho-GTP-ases that connects directly to targets like ITGB2 and ITGAM was upregulated, suggesting a subsequent increase in cellular adhesion and movement. Figure 2C (left panel) shows hierarchical clustering heatmaps, including NK cell signature markers (NCAM/CD56, killer receptors KLRK1 and KLRD1, IFNγ, IL-22, natural cytotoxicity receptor NCR1 and KIT stem cell factor). These markers shared similarity in their behavior based on Pearson correlation algorithms (Fig. 2C, right panel), and they were all upregulated in Long-COVID (Fig. 2C, lower panel). Figure 2D (top left panel) illustrates the pathway analysis scoring with TNFα as the top hit; this graph shows the enrichment p-value on the horizontal axis (in a negative log scale) and the perturbation p-value on the vertical axis [50].

Furthermore, pathway enrichment analysis demonstrated that TNFα signaling pattern is highly represented in plasma proteome of the Long-COVID, as opposed to heathy control subjects (next plot, top row, Fig. 2D). The remaining plots in Fig. 2D portrayed elevated ANGPT1, whereas ANGPT2 did not change suggesting an ANGPT1/ANGPT2 conversion phenomenon, as illustrated by the cartoon (Fig. 2D, bottom, right corner). The ANGPT1/2-Tie axis is likely a critical regulator of endothelial inflammation and vascular leakage [51–53], and excess of ANGPT1 may reduce this process. Potential ‘vascular perturbation’ in Long-COVID pivoting around ANGPT/VEGF axis was validated by ANGPT1 and VEGFA measurement in plasma provided by ELISA, in a different group of patients than those approached by targeted proteomics (Additional file 4: Fig S3, Immuno arrays analysis).

In addition to the NK phenotype shift, the percentual contribution of memory B cells, memory CD4 activated cells and neutrophils to plasma proteome appeared elevated in Long-COVID, whereas resting dendritic cells, CD4 memory resting cells and M1 macrophages seemed to be down-regulated (Fig. 2A).

### Immune cell resetting is potentially associated with vascular events mediated by VEGFA

In line with the results presented above, we observed that ANGPT1 and VEGF signaling also corelated with the TNFα pathway. Additional file 6: Fig S5A and Additional file 7: Fig S6 show that the VEGFA pathway could regulate endothelial cell migration (signaling map is a crop-out of the KEGG chart presented in Additional file 6: Fig S5, as an output of iPathwaysGuide software). Additional file 6: Fig S5B illustrates the upregulated markers that are members of the VEGFA pathway in Long-COVID, and included PXN, SRC, NOS3, and HSPB1 proteins. Other key markers such as Flt1 (VEGF receptor 1), AKT and MAPK, indicate endothelial cell survival, migration and proliferation. Key markers capable of protein–protein interactions are highlighted in the bottom left diagram that suggested VEGFA induced cell proliferation; a process that could be further mediated by SRC and MAP-Kinases, as well as AKT signaling to influence cell survival and/or migration.

### Plasma proteome analysis suggested “Neutrophil Extracellular Trap  formation”

**Fig. 3:**
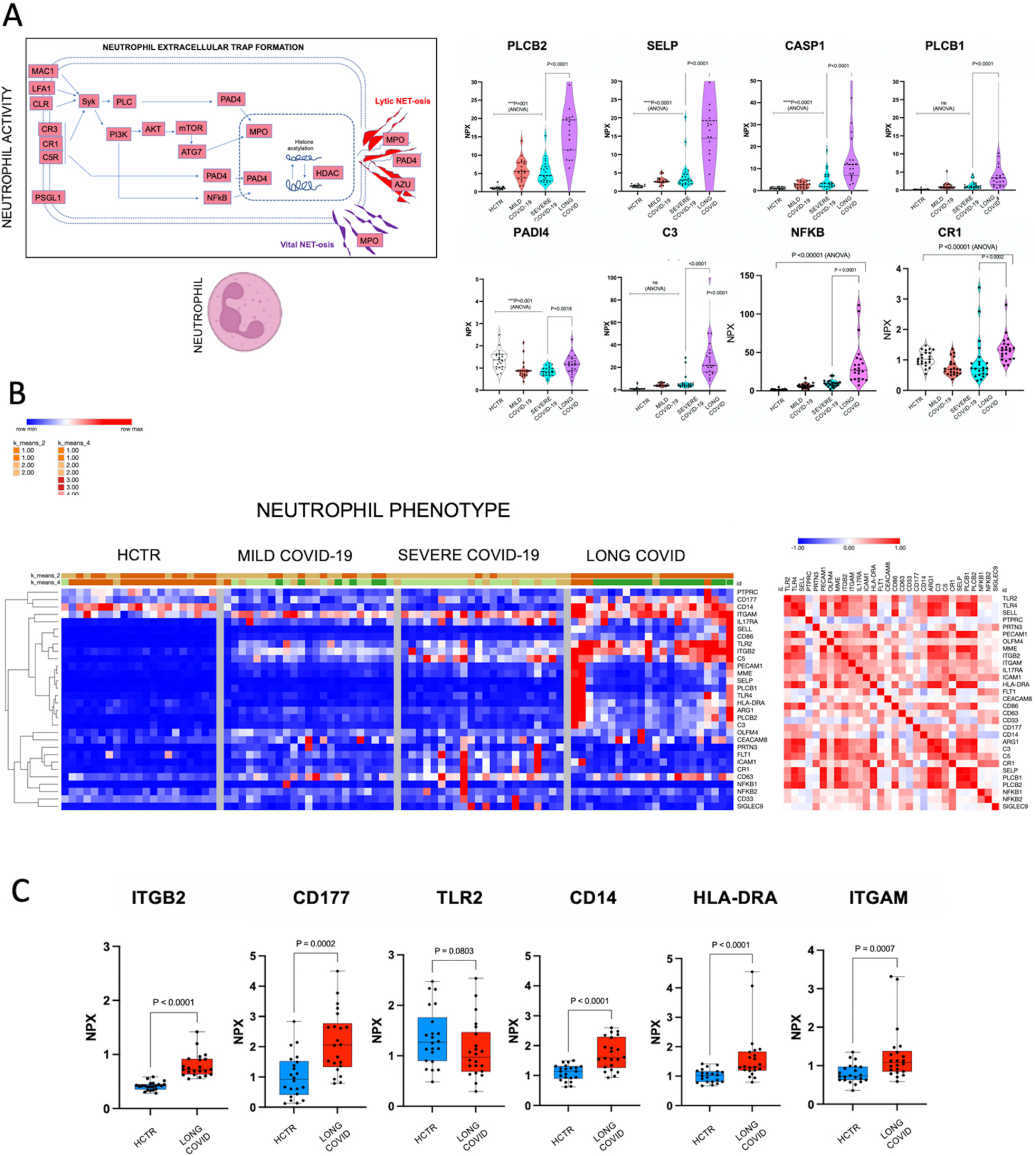
Neutrophil extracellular trap formation has a critical role in Long-COVID. **A** Diagram shows the NK cell cytotoxic pathways mapped on KEGG charts via iPathwayGuide platform. Up-regulated biomarkers are shown in red (KEGG Mapper, also confirmed by iPathwayGuide software). Graphs display the levels of individual NK-cell marker expression in plasma among the study cohorts. Statistical significance was determined with GraphPad 9, P-value was considered significant if < 0.05 with an ANOVA. **B** Heatmaps represent the profiling of the neutrophil cell phenotype. Markers have been manually curated. In the left panel, heatmap shows the hierarchical clustering of all markers and in the right panel the similarity matrix based on Pearson correlation. For data visualization we used Morpheus software from Broad Institute. **C** Graphs show individual neutrophil cell markers expression in plasma; comparison among all study cohorts. Statistical significance was processed with GraphPad 9, P-value was considered significant if < 0.05 with ANOVA

As shown in Additional file 8 Fig. S8, the features of neutrophil extracellular traps (NETs) include: (i) a defense mechanism against both micro-organisms and sterile triggers, (ii) a DNA scaffold with granule-derived proteins, such as proteases (e.g., elastase) or citrullinated histone H3, (iii) an important role in inflammation, autoimmunity and other pathophysiological conditions (either detrimental or beneficial), and/or (iv) it can be prompted by many triggers and via multiple distinct pathways with often unknown interrelationship [54–57]. The NET pathways shown in Fig. 3A represent a summary of the KEGG NET map output of iPathwayGuide, presented in Additional file 8: Fig S8. In red are the overexpressed biomarkers. Individual NET-specific/related biomarkers were featured in single expression graphs, where plasma phospholipase (PLC1 and PLCγ), CAS1, and PDA4 were significantly upregulated in Long-COVID, while NFkB, CR1, C3 and SLPG were depressed, compared to severe COVID-19. While these results appear conflictual, our group and others previously reported a repurposed neutrophil phenotype in severe COVID-19 that may be further differentiated in Long-COVID [54, 55].

Heatmaps representing the profiling of the neutrophil phenotype (based on a manually curated set of markers taken from specific literature reports) are presented in Fig. 3B [54–57]. The heatmaps show the hierarchical clustering of these markers in healthy control subjects and in COVID-19 patients along with their predicted inter-dependence (Pearson correlation algorithm; Fig. 3B, right panel). Figure 3C highlights increased expression of neutrophil based on CD177, HLA-DR, ITGAM, ITGB2 and TLR2 expression in Long-COVID, as compared to the other patient cohorts. Interestingly, the presence of CD14, which is an innate immunity marker produced by macrophages and neutrophils was similar in both healthy control subjects and Long-COVID patients, but significantly depleted in mild and severe COVID-19 patients. To validate NET formation in Long-COVID, we measured citrullinated histone H3 and elastase concentrations in plasma using  ELISA (results are presented in Additional file 5: Fig S4B). These markers are usually found in the neutrophil trap granules, with elevated citrullinated histone 3 being a specific marker for Long-COVID. A neutrophil cell pattern, along with several other cellular patterns (NK cell, microglia and astrocytes), are shown as heatmaps in Additional file 5: Fig S4C. These maps indicate the fluidity of certain cellular proteins present in plasma and they may indirectly indicate cellular status.

### EP/p300 could potentially favor vascular inflammation *via* TNF signaling

**Fig. 4:**
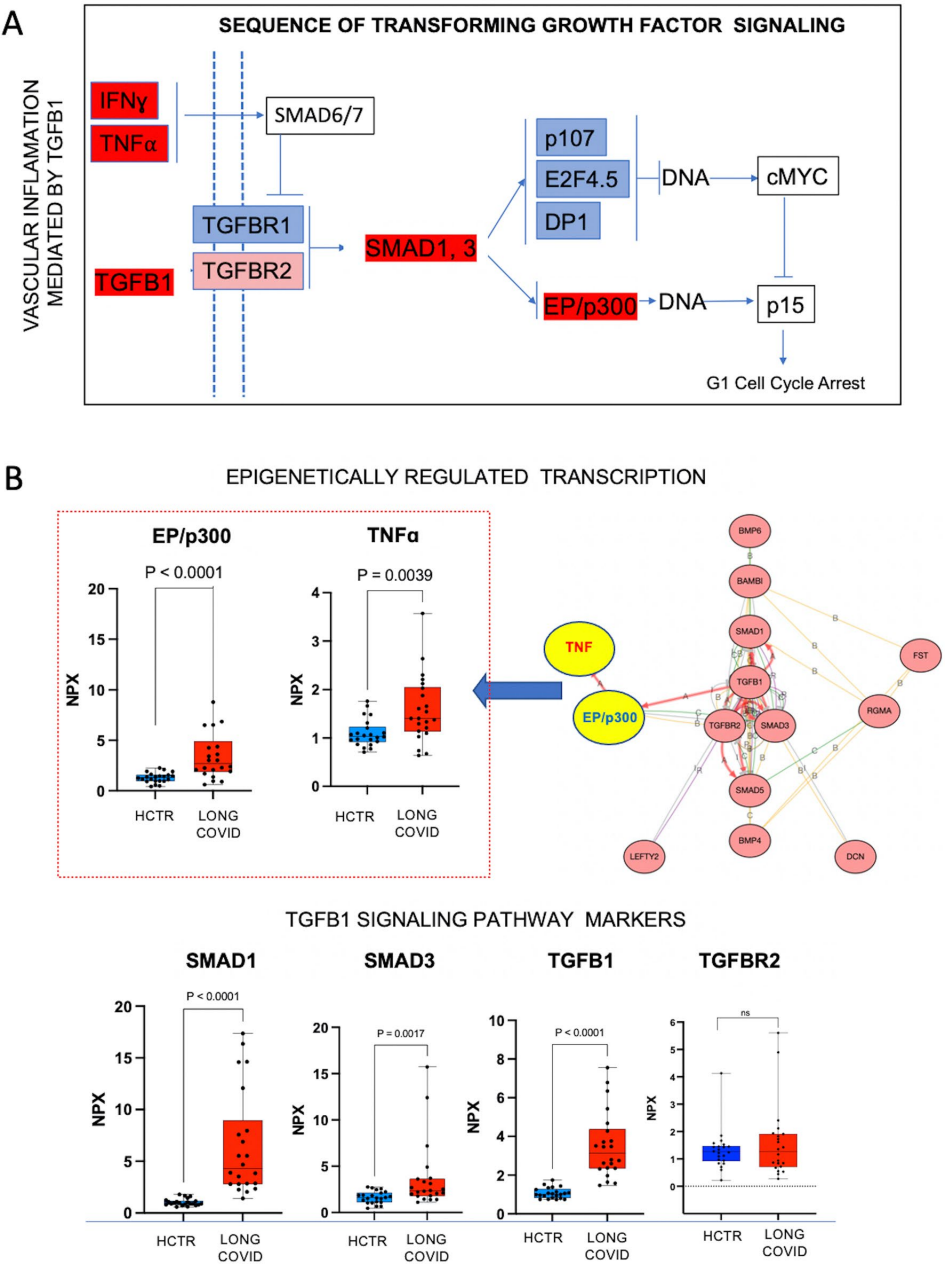
Increased expression of EP/p300 may repurpose TNF actions in Long-COVID. **A** Diagram shows TGFβ signaling pathways. Up-regulated biomarkers are shown in red and down-regulated in blue (KEGG Mapper, confirmed by iPathwayGuide software). **B** Graphs represent the expression levels of markers associated with the TGFβ1 pathway. Statistical significance was established using GraphPad-9, and P-value was considered significant if < 0.05 with Mann–Whitney U test. Key protein interactions are presented in the diagram from the middle panel (right side), an output of STRING confirmed with iPathwayGuide software. Fluctuation of the TGFβ and TNFα may impact EP/p300 epigenetic activity

Although the source of the plasma proteins, the fashion of their entry into the circulation, their life cycle, as well as their physiologic functions still remain largely matters of speculation, certain patterns with high statistical impact deserve close attention. Figure 4A illustrates TGFβ1 signaling pathway with both up-regulated (in red) and down-regulated (in blue) markers, per KEGG iPathwaysGuide output (Additional file 7: Fig S7). Figure 4B graphs represent the expression of individual markers associated with this TGFβ1 signaling pathway. Key marker interactions are presented in the diagram (upper right). The fluctuation of TGFβ1 and TGFBR2 allowed us us to speculate that such configuration may favor TNFα pro-inflammatory signaling to induce an acute form of glucocorticoid resistance (GCR) [56, 58, 59]. TNFα would have a significant and broad impact on the transcriptional performance of glucocorticoid receptor (GR), but no impact on nuclear translocation, dimerization, or DNA binding capacity of GR [59]. The GR cofactor that interacts significantly less with the receptor under GCR conditions is EP/p300, a plasma biomarker with high fluctuation in Long COVID that regulates the vascular bed *via* HIF, under hypoxic conditions. EP/p300 may strongly influence NFκB activation and thus, it could mediate inflammation [56]. Furthermore, it is known that EP/p300 knockdown reduces the transcriptional output of GCR, whereas its overexpression followed by NFκB inhibition reverts TNFα-induced GCR, trailing an authentic rearrangement model [59]. The extensive actions of EP/p300 include interactions with SMAD1 to bridge coactivators such as NFκB transcribing IL-6, or it may interact with HIF1α/VEGFA axis [6, 60] (Additional file 9: Fig S9).

### HIF signaling pattern reflected in the plasma proteome could be related to vascular proliferation

**Fig. 5:**
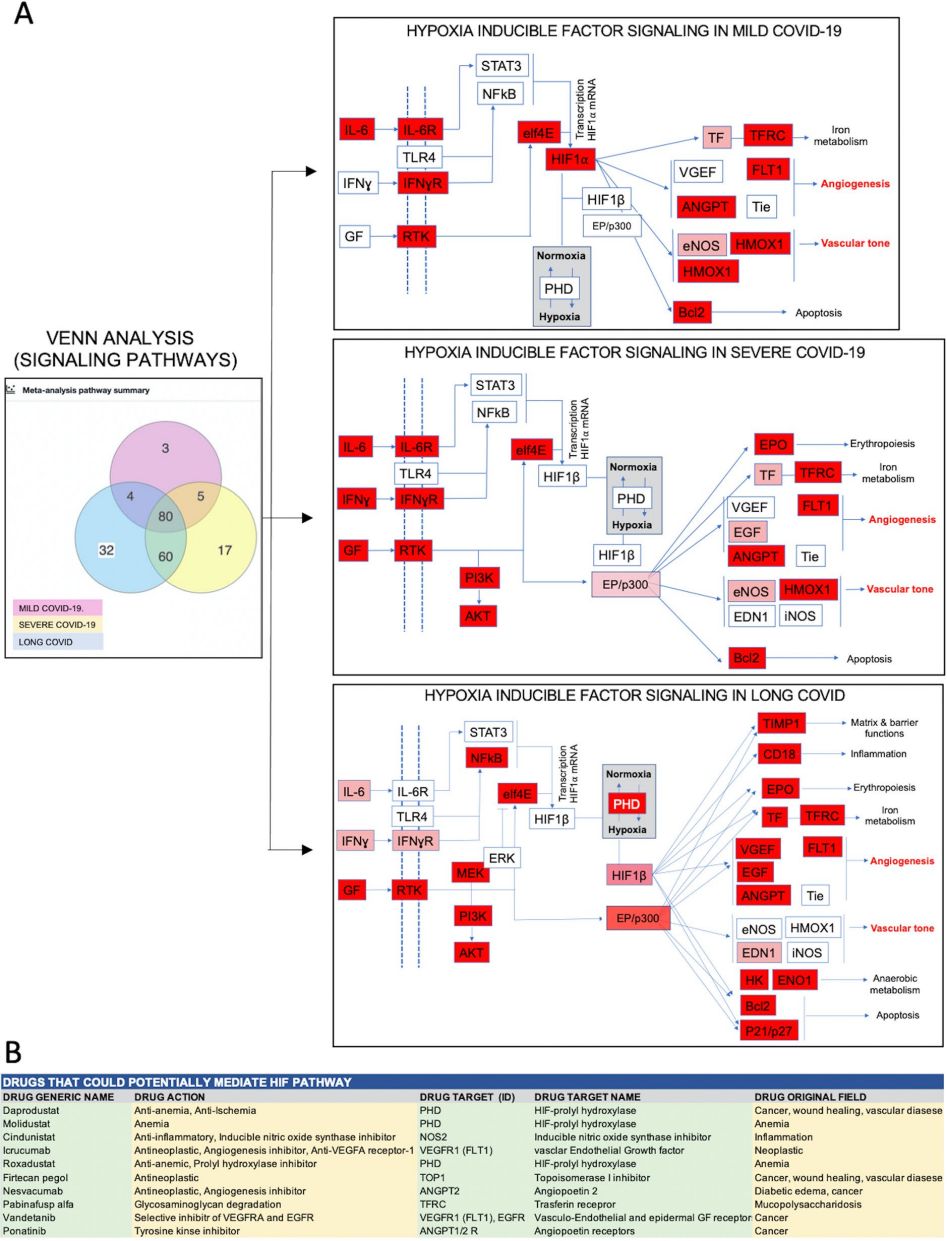
COVID-19 associated hypoxia is potentially mediated by HIF-1 and EP/p300, possibly disrupting the vascular bed. Long-COVID data has been intersected with Mild and Severe COVID-19 data sets, each normalized to the healthy control group (meta-analysis was performed with KEGG Mapper and confirmed by iPathwaysGuide software). Venn diagram at the left shows the intersection of the total number of signaling pathways among the clinical groups (iPathwayGuide). **A** One of the pathways predicted to support vasculo-proliferative disease was HIF-1-signaling pathway. Diagrams represent sequences of the HIF-1 pathways mapped onto KEGG charts and analyzed by iPathwayGuide software. The configuration of this pathway evolved from Mild COVID-19 to Severe COVID-19, having the most prominent representation in Long-COVID. Note: vasculo-proliferative disease is regularly mediated by HIF, which increases both proliferation and angiogenesis. **B** Table shows the drugs associated with the HIF pathway which can be repurposed for Long-COVID therapeutics (drug Bank output)

The Long-COVID proteome was intersected with the mild and severe COVID-19 data sets, as well as the healthy control group, in a meta-analysis performed by iPathwaysGuide software (Fig 5). In Fig. 5A (left diagram), the resulting Venn diagram shows that the Long-COVID cohort shared 80 signaling pathways among groups and 60 with severe COVID-19. Long-COVID still retains 32 unique (independent) pathways. Among all the pathways, HIF signaling was a major mechanism that was proportionally enriched with disease severity, to a maximum in Long-COVID, as seen in Fig 5A and Additional file 9: Figure S9 (where red arrows represent coherent cascades). Looking at the specific signaling map, certain molecular scenarios can be developed, where prospective HIF fluctuation in mild acute COVID-19 could be initiated by IL-6, and it may involve STAT3. Targets like ANGPT and FLT1, could also be affected by possible modifications in angiogenesis, iron metabolism (TFRC effector), vascular tone (HMOX1 effector), and cell survival (through BCL2 survival factor). In the severe COVID-19 cohort, HIF signaling could likely be triggered by IL-6, IFNγ and growth factors (VEGF and/or EGF) as ligands of receptor tyrosine kinases. Possible down stream modifications would be expected to include erythropoietin (EPO), angiogenesis (FLT1, EGF, ANGPT1), vascular tone (eNOS, HMOX1), aerobic metabolism (GAPDH, ENO1) and survival (BCL2). HIF activation, and its consequences, in Long-COVID seemed to be significantly affected by the simultaneous decrease in EP/p300, which suggested extracellular matrix consequences *via* TIMP1, CD18-dependent inflammation (Integrin β2, ITGB2), and Tie2 regulated angiogenesis. HIF is a leading regulator of hypoxic/ischemic vascular responses, driving transcriptional activation of genes involved in vascular reactivity, angiogenesis, and the deployment of bone marrow-derived angiogenic cells. In parallel, EP/p300 may function as a histone acetyltransferase with epigenetic functions reflected in endothelial cell proliferation and differentiation [6, 56, 58–61], perhaps contributing to epigenetic modifications induced by SARS-CoV-2 infection. EP/p300 may also affect the stimulation of hypoxia-inducible genes such as VEGF. While there are no therapeutic agents that specifically target EP/p300, it was predicted by the iPathwayGuide software that the HIF pathway can be regulated by multiple re-purposed drugs as presented in Fig 5B.

### Plasma proteome of Long-COVID reflects cell proliferation patterns

**Fig. 6:**
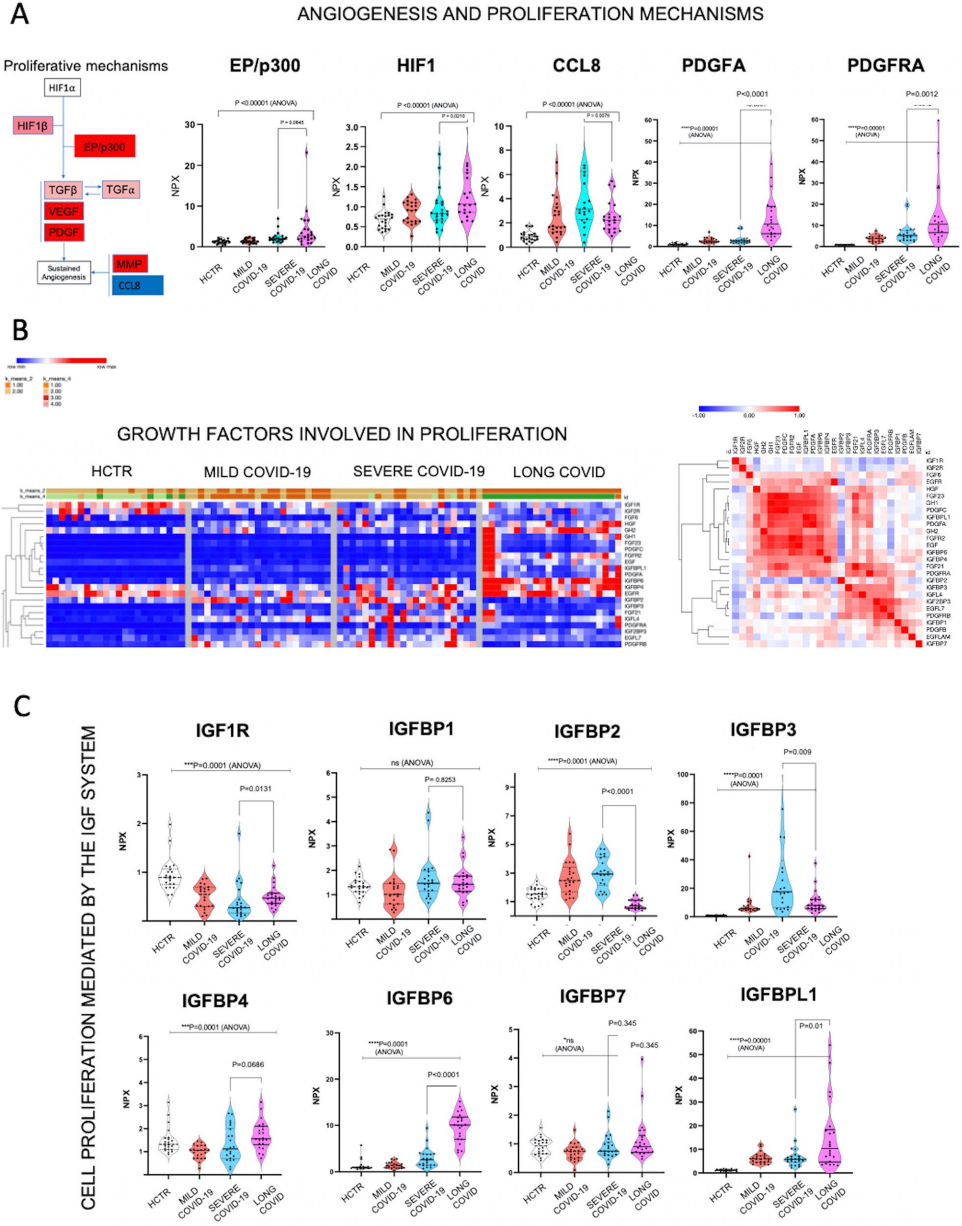
Long-COVID is associated with abnormal proliferation pathways, largely affecting the vascular bed. **A** Diagram at the left shows a sequence of proliferative signaling pathways mapped on KEGG charts (KEGG Mapper). The top right panel presents graphs depicting the expression of individual markers that belong to the proliferative pathways mediated by VEGF. Comparison has been done among all study groups and statistical significance was established using GraphPad-9, with P-value considered significant if < 0.05 with ANOVA. **B** Heatmaps reflect global expression levels of the growth-factors that could be responsible for the cell proliferation via receptor tyrosine kinases (RTKs) membrane receptors. Markers have been manually curated based on literature reports. In the left panel heatmap shows the hierarchical clustering of all markers and in the right panel the similarity matrix based on Pearson Correlation. For data visualization we used Morpheus software from Broad Institute. **C** Graphs measure the levels of the insulin-like growth factor system (IGF), molecules known to regulate angiogenesis but also to ensure the sustainability of the vascular network. Statistical significance was established using GraphPad-9, and P-value was considered significant if < 0.05 with ANOVA

To establish the nature of the vascular disease and its proliferative aspects, Long-COVID data was investigated using the KEGG cancer pathways (Additional file 10: Fig S10), with the most representative signaling cascade being HIF as highlighted in Fig. 6A (left panel). In this network, the up-regulated biomarkers are shown in red and the down-regulated shown in blue. Plots in Fig. 6A (right panel) illustrate markers elevated in Long-COVID. These proteins are usually responsible for the sustainability of the vascular bed. Figure 6B heatmaps reflect the levels of growth-factors that support cell proliferation through membrane receptor tyrosine kinases likely located within the vascular bed. These markers have been curated by the OLINK company. The diagram in the right panel of Figure 6B illustrates correlations between these factors in a similarity matrix based on Pearson correlation principles. Figure 6C graphs show the levels of the insulin-like growth factor (IGF) binding proteins that regulate the bioavailability of IGF, a factor known to regulate both angiogenesis and sustainability of the vascular bed. Except IGFBP4, the other IGFBPs are common to severe COVID-19. IGFBP-6 and IGFBPL1 seem to be Long-COVID specific. The status of the IGFs and their binding proteins could reflect the tissue repair capacity. A possible scenario is that the IGF system may combat the inflammatory events stimulating tissue and organ repair.

### Long-COVID associated brain dysfunction reflected in the plasma proteome

**Fig. 7:**
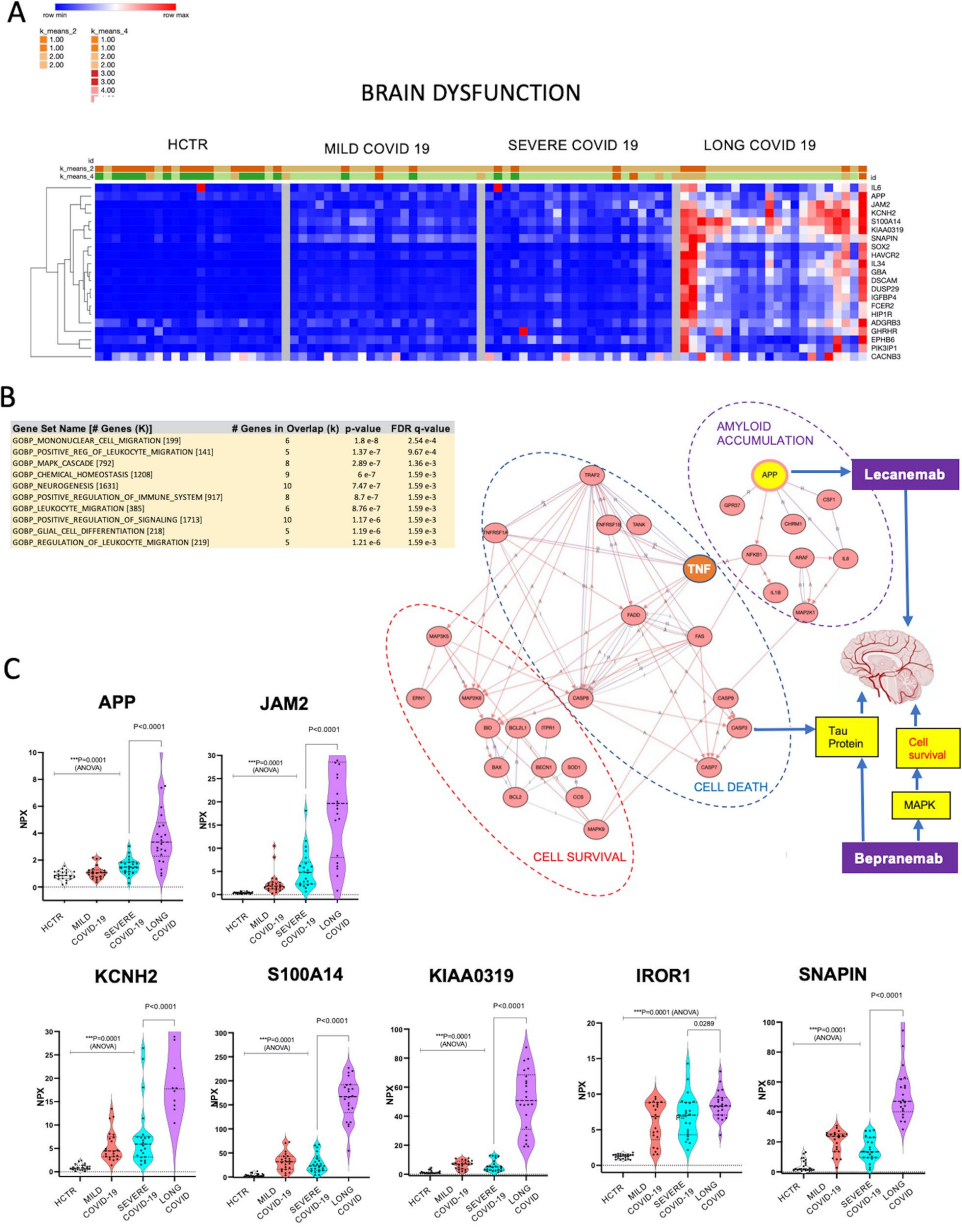
Long-COVID could impact brain functionality through vasculo-proliferative events possibly hosted by the blood–brain barrier. **A** Heatmaps reflect the levels of the most significant neurological markers per OLINK panels I, II and III, which were largely changed in Long-COVID patient plasma. Values were hierarchically clustered based on Pearson correlation algorithms. For data visualization we used Morpheus software, a tool designed by the Broad Institute. **B** Table shows functional annotation terms obtained with tools from the Gene Set Enrichment Analysis (GSEA) platform. These clusters refer to leucocyte migration, positive immune signals, glial cell differentiation, neurogenesis and MAPK regulatory pathways. At the right, diagram shows a segment of brain degeneration pathways as described by KEGG charts and processed for protein–protein interaction capacities with STRING software (confirmed by iPathwayGuide). TNF and APP proteins are highlighted as major players. The latter finding suggests that Lecanemab, a humanized IgG1 monoclonal antibody that targets amyloid beta, could be considered as a disease modifying immunotherapy. According to KEGG encyclopedia (target-based classification of drugs chapter), Bepranemab is a humanized, monoclonal antibody that binds to the central region of the Tau protein whose primary role is maintenance of the microtubules in neuronal axons. Bepranemab affects three target pathways in the brain: i) Neurodegeneration, ii) Alzheimer Disease, and MAPK pathways related to cell survival. Taken together, this data is indirectly pointing to a possible blood brain barrier dysfunctionality based on cell proliferation. **C** The expression levels of markers from the above functional groups have been plotted on graphs, where comparison has been done among all study groups. P-value was considered significant if < 0.05 with ANOVA. One highly expressed biomarker marker was the Amyloid Precursor Protein (APP/Presenilin) that is mainly known as a pathognomonic marker for Alzheimer disease and/or brain inflammation

The neurologic manifestations of the COVID-19 are well characterized and a comprehensive evaluation of the post-acute neurologic sequelae at 1 year was recently undertaken [62–65]. COVID-19 increased the risk of numerous neurologic sequelae such as ischemic and hemorrhagic stroke, cognition and memory disorders, peripheral nervous system disorders, episodic disorders (migraine and seizures), extrapyramidal and movement disorders, mental health disorders, musculoskeletal disorders, sensory disorders, Guillain–Barré syndrome, encephalitis, and/or encephalopathy. In Fig. 7A, hierarchical clustering heatmaps reflect the levels of neurological markers across the patient groups (curated by OLINK). Expression levels were hierarchically clustered in unsupervised heatmaps based on Pearson correlation algorithms. Markers selected through the above methodology were investigated for functional annotation using tools from the GSEA platform and MSigDB data repositories (Fig. 7B). This latest analysis demonstrated that functional clusters were formed around leukocyte migration, positive immune signals, glial cell differentiation, neurogenesis and MAPK regulatory modules. Taken together, these pathways suggest dysfunction of the brain-blood barrier grounded on cell proliferation. Graphs in Fig. 7C illustrate the expression levels of individual markers from the functional groups presented in Fig. 7B. One of the highly expressed markers, was the amyloid precursor protein (APP) which is known to be a pathognomonic marker for both Alzheimer disease and brain inflammation [62–66]. Additional markers for brain dysfunction include JAM2 (endothelial tight junctions protein), SNAPIN (a mediator of neuronal autophagy-lysosomal function in developing neurons), KCNH2 (potassium channel), S100A14 (involved in cell motility adhesion and growth), KIAA0319 (language impairment biomarker), and IROR1 (a receptor tyrosine kinase like orphan receptor 1, which regulates neurite growth in the central nervous system, mediates WNT-signaling and maintains the auditive apparatus).

### Long-COVID associated cardiometabolic dysfunction reflected in the plasma proteome

**Fig. 8:**
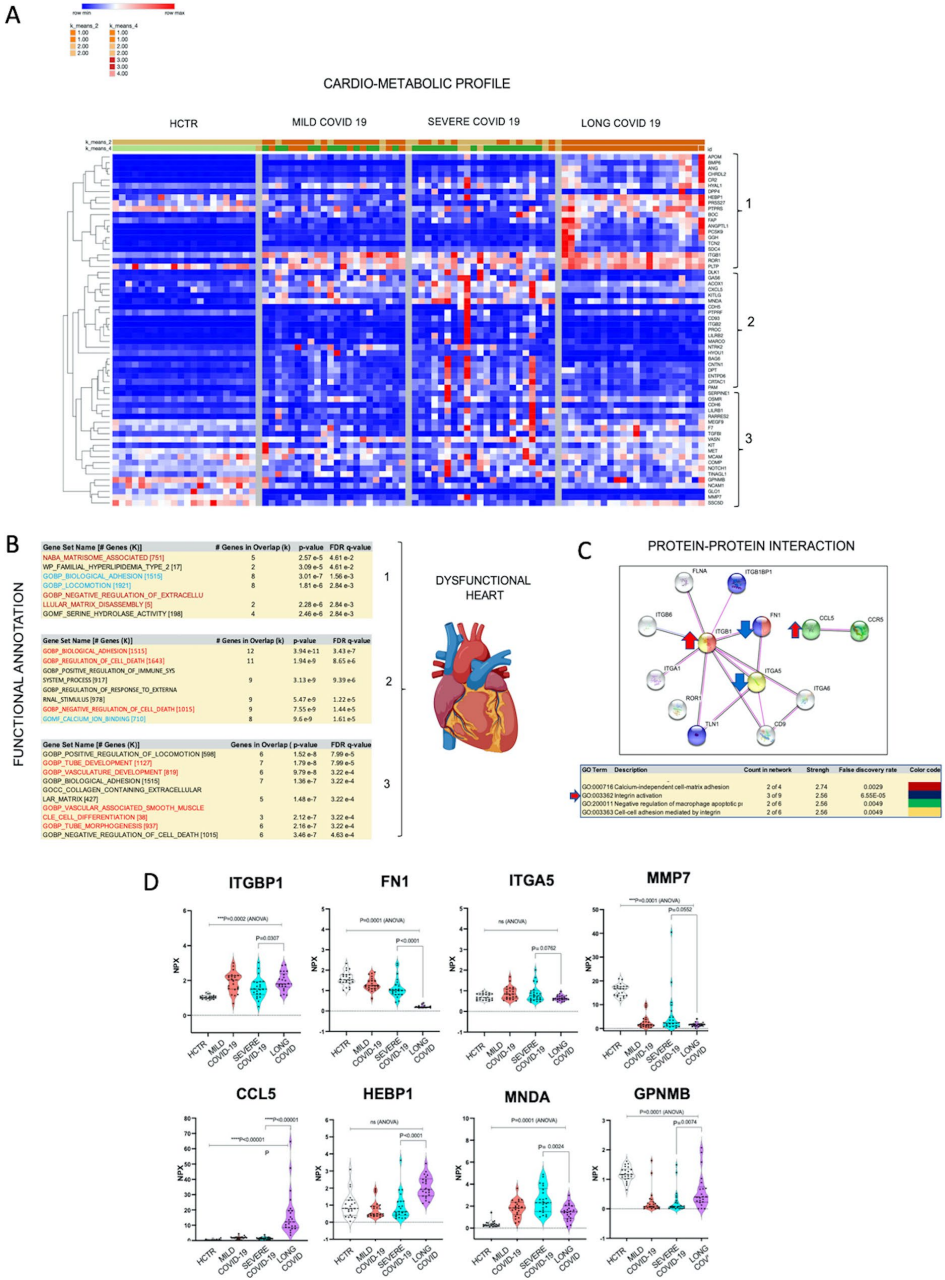
Long-COVID is potentially associated with cardiometabolic damage caused by vasculo-proliferative events. **A** Heatmaps reflect the levels of the most significant cardio-metabolic markers (per OLINK panels I and II) that were changed in Long-COVID patients. Markers have been curated by OLINK. Values have been hierarchically clustered based on Person correlation algorithms. For data visualization we used Morpheus software, a tool designed by the Broad Institute. **B** Post-hierarchical analysis these markers formed three functional clusters (#1, 2 and 3) determined with tools from the GSEA platform. These clusters refer to extracellular matrix remodeling, cell adhesion and motility, and angiogenesis (possible tube formation). **C** Markers forming the three functional clusters were analyzed for protein–protein interaction using STRING software (Search Tool for the Retrieval of Interacting Genes/Proteins). This analysis shows that extracellular matrix remodeling is dominated by integrin interactions and calcium binding events, as well as dysregulated macrophage activity (also observed by CIBERSORT analysis). **D** The expression levels of the markers that interact directly was plotted on graphs, where comparison has been done among all patient cohorts and statistical significance was determined by GraphPad-9 (P-value was considered significant if < 0.05 with ANOVA)

COVID-19 is associated with long-term cardiac dysfunction [6, 66]. In Fig. 8A, hierarchical clustering heatmaps reflect the levels and the dynamics of cardio-metabolic markers across all patient cohorts. Markers were curated by OLINK and their expression levels were hierarchically clustered using Morpheus software tools. Topographically, three critical clusters were formed (Fig. 8B) by standard screening using GSEA/MSigDB tools. The three clusters could also be functionally annotated; taken in order from 1 to 3, clusters refer to (i) extracellular matrix remodeling, (ii) cell adhesion and motility and (iii) angiogenesis (tube formation). These functional clusters were then investigated for protein–protein interactions as presented in Fig. 8C. In this configuration, the extracellular matrix remodeling and cell–cell adhesion functions were dominated by integrins (ITGB1, ITGA5, ITGA1, ITGB6, ITGB1B1), which can potentially interact with fibronectin (FN1), filamin (FLNA) and calcium binding markers, essentially mediating Ca^2+^-independent—cell matrix interactions. CCL5 was highly elevated and usually mediates the HIF-1α pathway during hypoxia, chronic inflammation and angiogenesis [67]. Graphs in Fig. 8D represent individual marker expression levels in plasma. The integrin changes depicted in these graphs may result in a disbalanced extracellular-matrix, possibly leading to apoptosis of endothelial cells.

## Discussion

SARS-CoV2 infection has proclivity for long term disease, with profound impact on brain and heart (Additional file 11: Fig S11). To elucidate potential mechanisms, the plasma proteome of Long-COVID patients was investigated as a surrogate of cell/tissue activities; the plasma proteome was deconvoluted into different cell types and signaling mechanisms, as compared to age- and sex-matched acutely ill COVID-19 inpatients and healthy control subjects. Individual biomarker expression was also analyzed among the patient cohorts to identify potential value in diagnostic and prognostic targets. The source of the plasma proteins, the fashion of their entry into the circulation, their life cycle, as well as their physiologic functions still remain largely matters of speculation [68, 69]. But, despite these caveats, we analyzed the plasma proteome using a novel immunoassay with NGS technology, followed by complex bioinformatic analysis. The latter was useful for organizing plasma proteins into patterns that mirror biological processes, signaling pathways, cellular components and cell type contributions to plasma content.

Our analyses of Long-COVID suggest that NK cells could potentially switch their phenotype from an activated to resting state and neutrophils appear predisposed to extracellular trap formation. These findings were validated with quantitative immunoassays. NK-redistribution was estimated previously by the expression levels of CD56 vs CCR7 [70], while neutrophil trap formation has been characterized by elevated plasma elastase and citrullinated histone H3. The cell-type contributions to plasma proteome, and their predicted phenotypes, were reflected in vascular-proliferation patterns where events could be mediated by TNFα, ANGPT1, VEGFA, TGFβ1, and EP/p300. In this analytic scenario, the vasculo-proliferative state can be potentially anchored to HIF-1 pathways. Concurrently, the fluctuations of EP/p300 could influence HIF-1 function in Long-COVID. EP/p300 is a large protein with multiple cellular functions, including stem cell effector efficacy; thus, EP/p300 plays a major role in the reprogramming events leading to a proliferative phenotype with the acquisition of drug resistance and cell plasticity [71]. EP/p300, as a part of various transcriptional complexes, can alter critical biological functions such as cellular proliferation, cell cycle regulation, apoptosis, DNA damage repair, cell fate determination and stem cell pluripotency [71, 72].

**Fig. 9:**
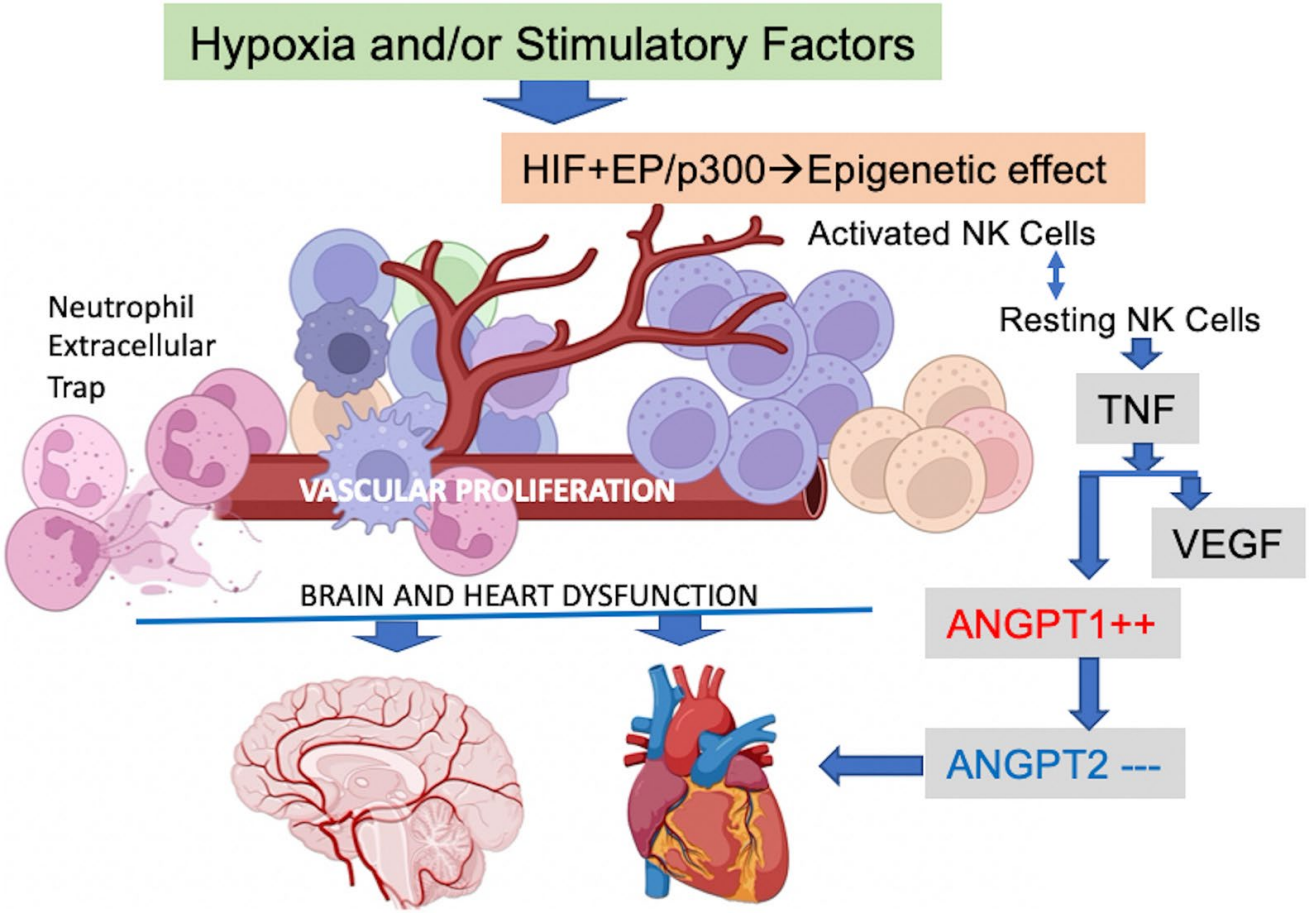
Schematic summary of the Long-COVID pathology as reflected in plasma proteome

Our study, using the plasma proteome as a surrogate of cell/tissue activities, may provide an insight with regards to Long-COVID pathophysiology and potential preventative strategies (Fig. 9). ANGPT1 has been shown to be up-regulated in Long-COVID [4, 5] and this protein plays an important role in vascular development and angiogenesis *via* endothelial tyrosine-protein kinase receptor (TEK/Tie receptor) [53]. ANGPT1 may be a critical compensatory protein in Long-COVID, mediating reciprocal interactions between endothelium and the surrounding extracellular matrix (ECM), interacting with the mesenchyme to inhibit endothelial permeability, while sustaining angiogenesis, endothelial cell survival, proliferation, motility and vascular quiescence [53]. In quiescent vessels, ANGPT1 usually forms complexes with TEK kinases from contiguous cells, leading to preferential activation of phosphatidylinositol 3-kinase and the AKT1 signaling cascades. Migrating endothelial cells that lack cell–cell adhesion capacity, rely on ANGT1 to recruit TEK and influence the ECM, resulting in the formation of focal adhesion complexes. However, their interactions with ECM could be impaired in Long-COVID due to down-regulated MMP7, thereby reducing migration and tissue repair. Moreover, depressed MMP7 would impede the proteolytic release of TNF from macrophages, which is typical for hypoxia, and the release of VEGF from its receptor (VEGFR1/Flt1) [73–75] Elevated ANGPT1 in Long-COVID might be compensatory to the above alterations, thereby contributing to the activation of PTK2/FAK and downstream kinases MAPK1/ERK2 and MAPK3/ERK1; events that ultimately stimulate angiogenesis and blood vessel maturation. Hypoxic conditions occurring in acute COVID-19 could induce growth factors such as VEGF, PDGF, EGF and IGF, ultimately potentiating cell proliferation in the vascular bed, and may hold true for those patients with “happy hypoxia” (or local tissue hypoxia) [76, 77]. Alternatively, ANGPT1 may be a critical link between the VEGFA, HIF and TNFα signaling pathways that were impacted in Long-COVID. VEGFA, for instance, could play a crucial role in mediating reciprocal interactions between the endothelium and surrounding matrix and mesenchyme, due to the ECM remodeling detected in Long-COVID [4, 6, 53, 56, 58–61].

Our data also suggest that Long-COVID has the potential to severely impact the functionality of multiple organs *via* these aforementioned vasculo-proliferative actions. Differential protein expression and network analysis showed disruption of tissue recovery mechanisms that could be directly related to concerted HIF, TNFα and VEGFA signaling actions linked by ANGPT1. Besides providing system-level insights into the mechanism of Long-COVID pathology, our study identified potential biomarkers and therapeutic strategies that might be tailored to specific immune and organ mechanisms to combat the initial effects of  hypoxia.

Consistent with our findings, alternatively polarized macrophages were a major contributor to Long-COVID induced molecular alterations [78]. In addition, an anti-inflammatory profile was observed in Long-COVID patients based on insignificant levels of acute phase (IGFγ, IL-1) and macrophage-derived proteins (IL-18, MCP1, sTNFRII) [78].

High expression levels of the APP antigen were measured in plasma from Long-COVID outpatients. The APP gene encodes a cell surface receptor and transmembrane precursor protein that is cleaved by secretases to form a number of peptides, some of which are secreted and can bind to the acetyltransferase complex to promote transcriptional activation, while others form the protein basis of the amyloid plaques found in the brains of patients with Alzheimer disease [62–65]. Mutations in this gene have been implicated in autosomal dominant Alzheimer disease and cerebro-arterial amyloidosis (cerebral amyloid angiopathy). Amyloids were shown to be highly toxic to neuronal cells [62–65]. In this context, and due to the high amounts detected in plasma, our findings suggest that cytotoxic aggregates may be associated with long term neurological symptoms in Long-COVID.

Cardiometabolic changes were suggested by in Long-COVID plasma profiles, based on integrin signaling. ITGBP1 and INGA5 seem to engage FN1, to regulate focal adhesion (FAK)-related apoptosis pathways. Elevated CCL5 could maintain an inflammatory state during Long-COVID and together with resting NK cells that express CCR5 [77], promote angiogenesis. The latter via PI3K/AKT, NF-κB, HIF, RAS-ERK-MEK, JAK-STAT and TGFβ-SMAD pathways [78, 79]. However, these mechanisms would be likely independent of the myeloid lines and their inflammatory actions since the myeloid marker MNDA was up-regulated in Long-COVID. Furthermore, the increased levels of HEBP1 suggests a vascular proliferative disease, as this protein includes a natural chemoattractant peptide acting as a natural ligand for formyl peptide receptor-like receptor 2 (FPRL2). The FPRL2 receptor promotes calcium mobilization and chemotaxis in monocytes and dendritic cells, potentially instigating myocarditis [76, 77, 79]. FPRL2 peptides are powerful neutrophil chemotactic factors and activators [76, 77, 79], and possibly instigate neutrophil trap formation. Dysregulated inflammation can potentiate maladaptive healing and pathological remodeling of cardiac tissue, leading to long term cardiac dysfunction, features sometimes present in Long-COVID. As preclinical studies support the use of FPR2 agonists in heart disease, these therapeutic agents may be applicable for the prevention and treatment of cardio-metabolic pathology in Long-COVID [79].

Our study has unveiled potential cell-type signatures and signaling pathways in Long-COVID; however, there are several study limitations worthy of discussion. First, we investigated a limited number of patients, which based on the timing of their infection and data from our regional public health surveillance program, would have been infected with the wild-type or alpha SARS-CoV-2 variants. It is currently unknown whether all variants of concern would result in a similar pattern of symptoms and underlying pathophysiology. Second, we deconvoluted plasma proteins with sophisticated software to determine cell-type signatures, thereby providing information on cell type contributions to the plasma profiles. Future studies should evaluate cell numbers in fresh blood samples with flowcytometry-based technologies. Third, the plasma proteome was investigated as a surrogate for cell/tissue activities, and when combined with bioinformatics, identified signaling pathways for further investigation. Analyses of the plasma proteome seems reasonable given the broad pathology of this systemic illness, and the practical limitations of obtaining multiple human tissues. Nonetheless, our conclusion must be tempered by the lack of understanding of protein release and turnover. Finally, key signaling pathways were identified, but may still be missing key biomarkers as the proteomics approach used herein was targeted, and not all pathway constituents were available for measurement Fig. 9.

In conclusion, our report provides a pathophysiological framework to better understand the functional heterogenicity of Long-COVID and provides clues to the neurological and cardio-metabolic basis of this disease. This study also provides a valuable resource for the exploration of biomarkers in Long-COVID and the development of potential therapeutic targets for its prevention and/or treatment based on the potential pathophysiological mechanisms unveiled.

## Supplementary Information

**Additional file 1:** Long-COVID Outpatient Demographics and Clinical Data.

**Additional file 2:** Acutely ill COVID-19 Inpatient Demographics and Clinical Data.

**Additional file 3:** **Figure S1.** Study model and dimensionality reduction of data sets including all patients.Model in the left panel suggests three types of Covid-19 disease data setsrun against a healthy control group. Data represents plasma proteome obtained by Proximity Extension Assay, the average counts for all patients of each group. Right panel informs on type of data processing.Principal components analysis and hierarchical clustering. Unit variance scaling was applied to rows; SVD with imputation is used to calculate principal components. X and Y axis show principal component 1 and principal component 2 that explain 27.5% and 15.1% of the total variance, respectively. N = 88 data points. Data was processed by Clustvis software.

**Additional file 4:** **Figure S2.** Natural killer cell mediated cytotoxicity map. **Figure S3.** Serological validation of Angiopoetin-1 and Vasculo-Endothelial Growth Factor A. Each block in the heatmaps represent the mean of three technical replicates. Markers were measured in human plasma using a custom multiplexed immunoassay kit according to manufacturer’s instructions.

**Additional file 5:** **Figure S4.** Cell typing validation. Graphsandrepresent NK cell and respectively Neutrophil Trap Formationvalidation by ELISA. Plasma was collected from different patient cohorts from those analyzed by targeted proteomics. Two markers of each cell type were chosen as follows: NK cell phenotype was validated by analysis of CD56/NCAM and CCR7 markers emphasizing silent vs activatedNK cells; NET phenotype was analyzed by estimation of abundance of Elastase and Citrullinated Histone 3 in plasma, two markers that are usually found in the NET trap granules. Data was process by GraphPad 9, and significance was considered from P<0.05. Samples were processed and analyzed in three technical replicates.

**Additional file 6:** **Figure S5.** Resetting of cell type-associated protein abundance patterns in plasma may reflect vascular events mediated by VEGFA.VEGF signaling pathways as described by the KEGG are presented. Up-regulated biomarkers are in red and down-regulated biomarkers in blue. Analysis was done with KEGG Mapper and confirmed with iPathwayGuide software.Graphs show individual marker expression in Long-COVID plasma, as compared to healthy control subjects. Statistical significance was established using GraphPad-9, and P-value was considered significant if <0.05 in Mann-Whitney U test. Key markers were VEGFA, VEGFR2, AKT and MAPK, indicating endothelial cell activity, survival and migration. Key markers protein-protein interactions are presented in the diagram from the bottom panel, left.

**Additional file 7:** **Figure S6.** VEGF Signaling pathway. **Figure S7.** TGFB1 Signaling pathway.

**Additional file 8:** **Figure S8.** Neutrophil Extracellular Trap Formation.

**Additional file 9:** **Figure S9.** HIF1 Signaling pathways resulted from the pathways meta-analysis between HCTR, mild COVID-19, severe COVID-19 and Long-COVID groups.

**Additional file 10:** **Figure S10.** Cancer pathway and biomarkers that appear in Long-COVID patient plasma.

**Additional file 11:** **Figure S11.** Neurological dysfunction pathway and biomarkers that appear in Long-COVID patient plasma.

## Data Availability

By request.
