# Supplementary figures and images for: Plasma proteome of Long-COVID patients indicates HIF-mediated vasculo-proliferative disease with impact on brain and heart function
Source: J Transl Med. 2023 Jun 10;21:377. doi: 10.1186/s12967-023-04149-9 (PMC10257382; doi:10.1186/s12967-023-04149-9)

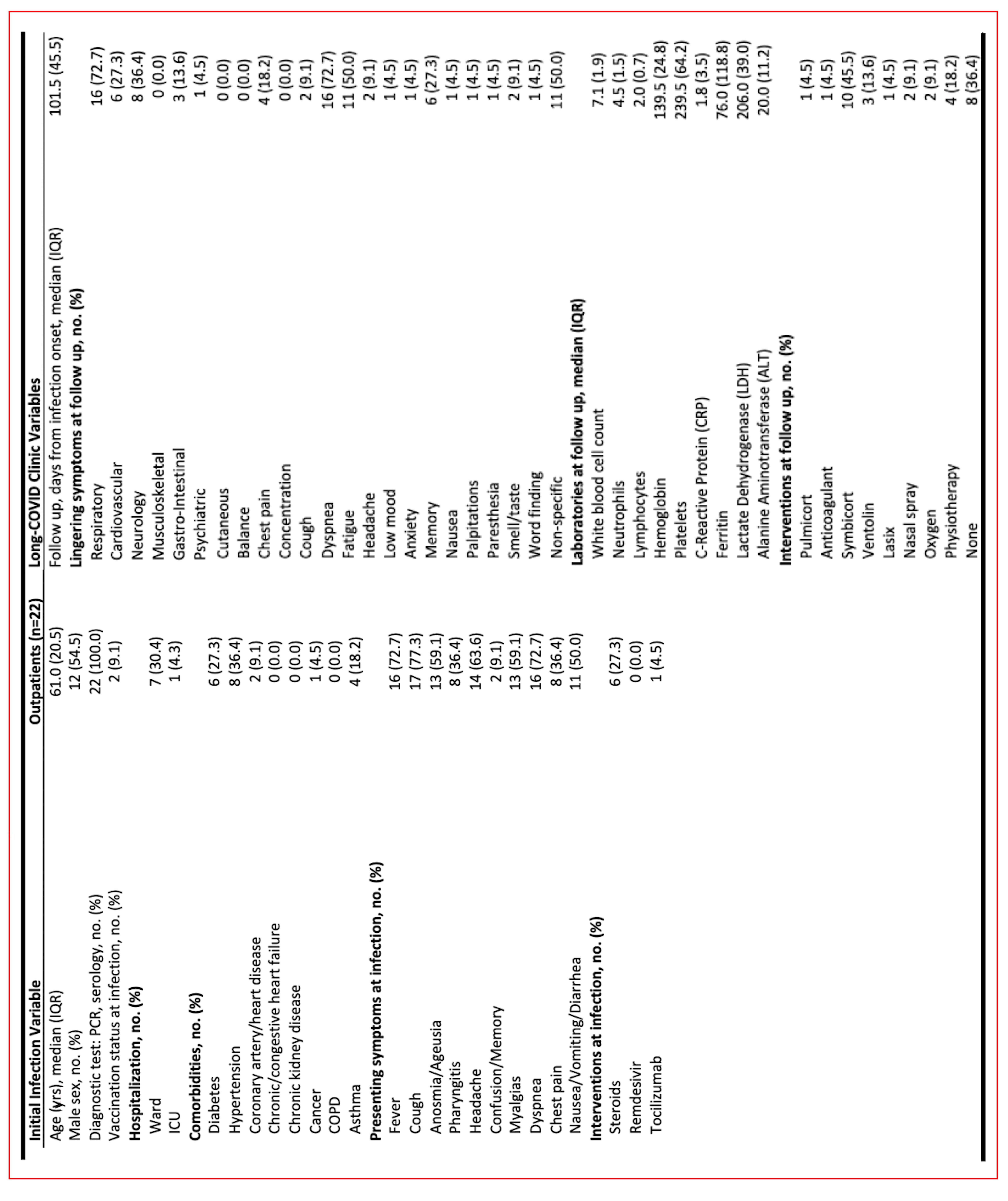

Supplement: Supplementary file 1 — Additional file 1: Long-COVID Outpatient Demographics and Clinical Data. [file 12967_2023_4149_MOESM1_ESM.jpg]

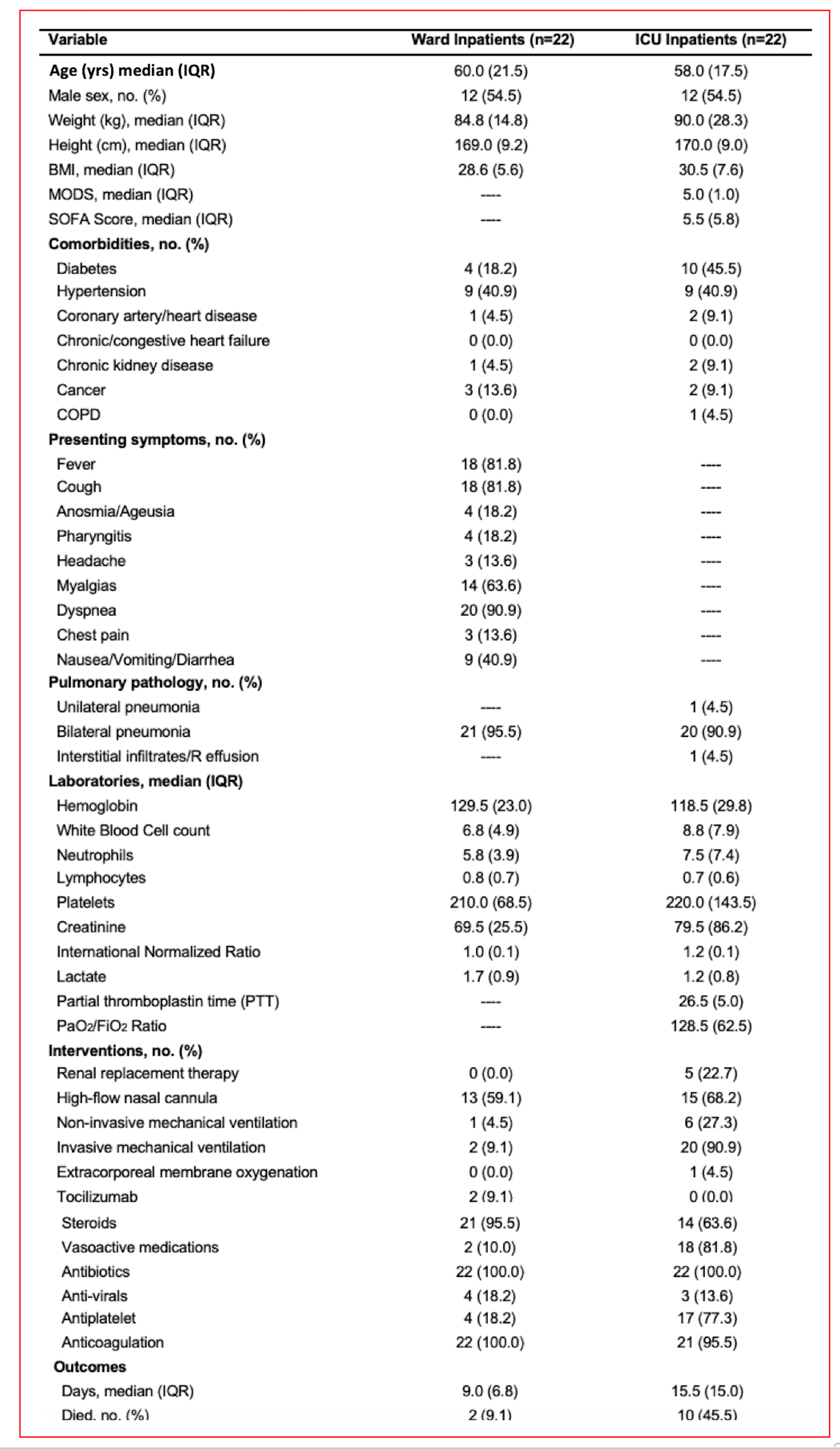

Supplement: Supplementary file 2 — Additional file 2: Acutely ill COVID-19 Inpatient Demographics and Clinical Data. [file 12967_2023_4149_MOESM2_ESM.jpg]

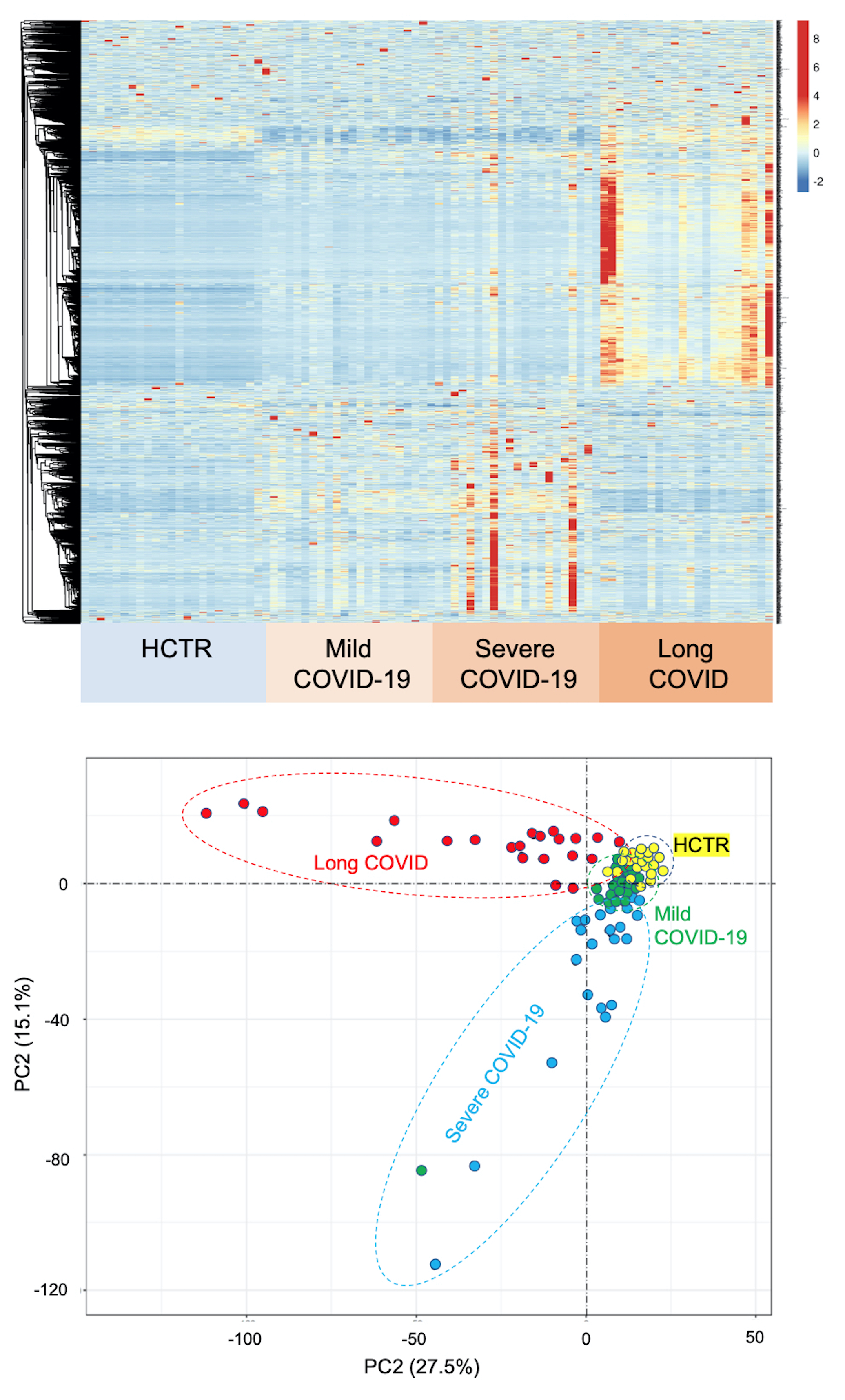

Supplement: Supplementary file 3 — Additional file 3: Figure S1. Study model and dimensionality reduction of data sets including all patients.Model in the left panel suggests three types of Covid-19 disease data setsrun against a healthy control group. Data represents plasma proteome obtained by Proximity Extension Assay, the average counts for all patients of each group. Right panel informs on type of data processing.Principal components analysis and hierarchical clustering. Unit variance scaling was applied to rows; SVD with imputation is used to calculate principal components. X and Y axis show principal component 1 and principal component 2 that explain 27.5% and 15.1% of the total variance, respectively. N = 88 data points. Data was processed by Clustvis software. [file 12967_2023_4149_MOESM3_ESM.jpg]

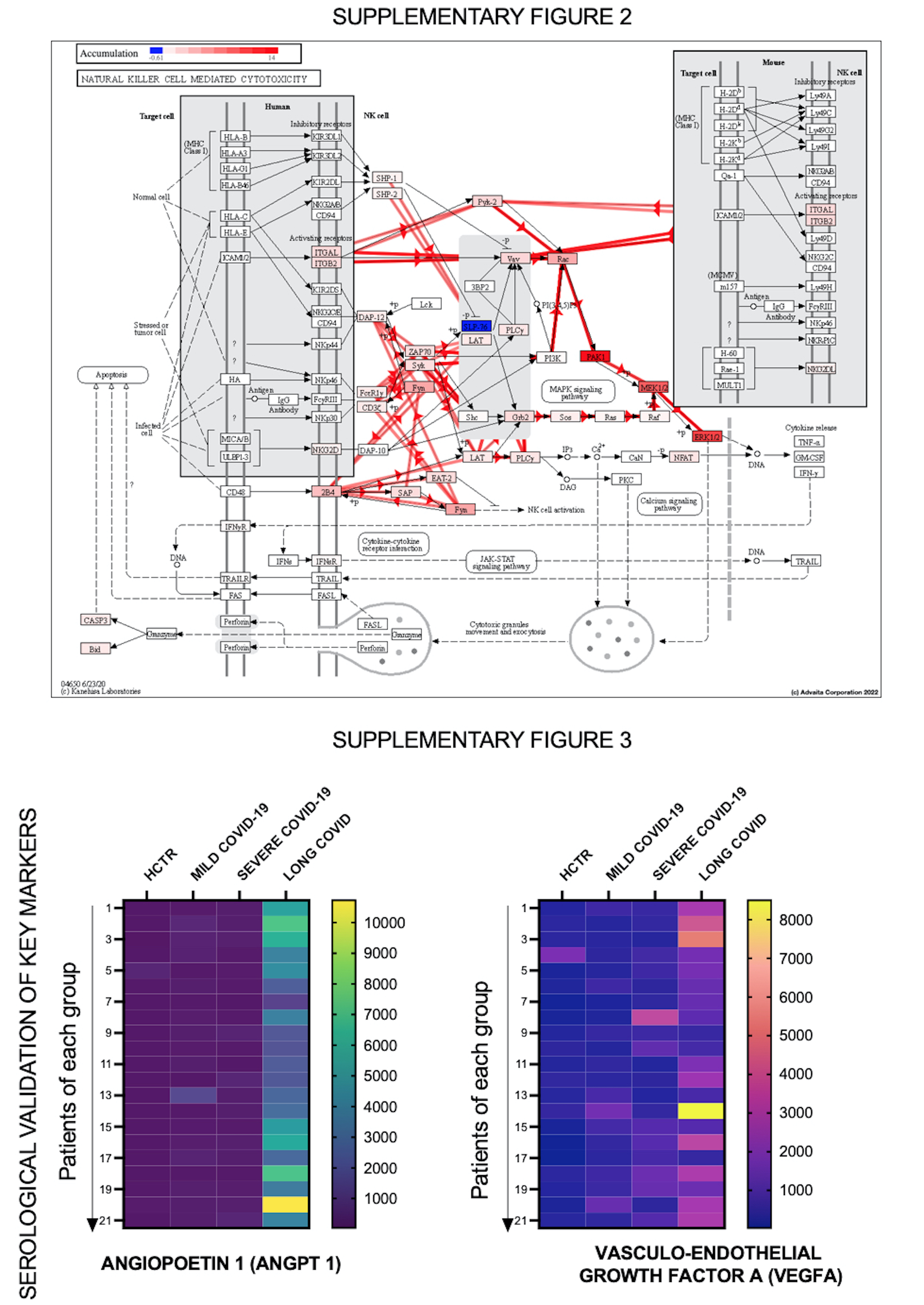

Supplement: Supplementary file 4 — Additional file 4: Figure S2. Natural killer cell mediated cytotoxicity map. Figure S3. Serological validation of Angiopoetin-1 and Vasculo-Endothelial Growth Factor A. Each block in the heatmaps represent the mean of three technical replicates. Markers were measured in human plasma using a custom multiplexed immunoassay kit according to manufacturer’s instructions. [file 12967_2023_4149_MOESM4_ESM.jpg]

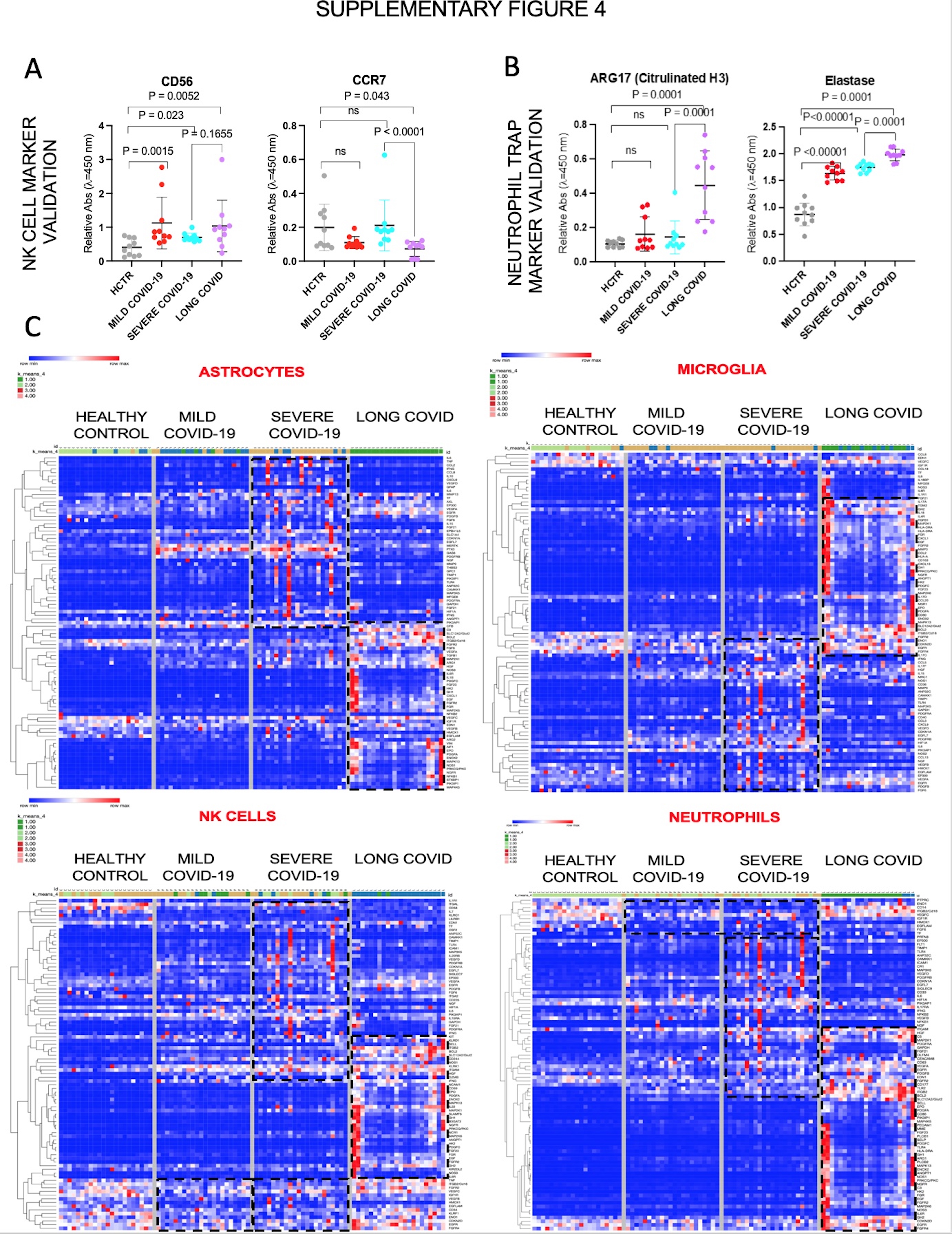

Supplement: Supplementary file 5 — Additional file 5: Figure S4. Cell typing validation. Graphsandrepresent NK cell and respectively Neutrophil Trap Formationvalidation by ELISA. Plasma was collected from different patient cohorts from those analyzed by targeted proteomics. Two markers of each cell type were chosen as follows: NK cell phenotype was validated by analysis of CD56/NCAM and CCR7 markers emphasizing silent vs activatedNK cells; NET phenotype was analyzed by estimation of abundance of Elastase and Citrullinated Histone 3 in plasma, two markers that are usually found in the NET trap granules. Data was process by GraphPad 9, and significance was considered from P<0.05. Samples were processed and analyzed in three technical replicates. [file 12967_2023_4149_MOESM5_ESM.jpg]

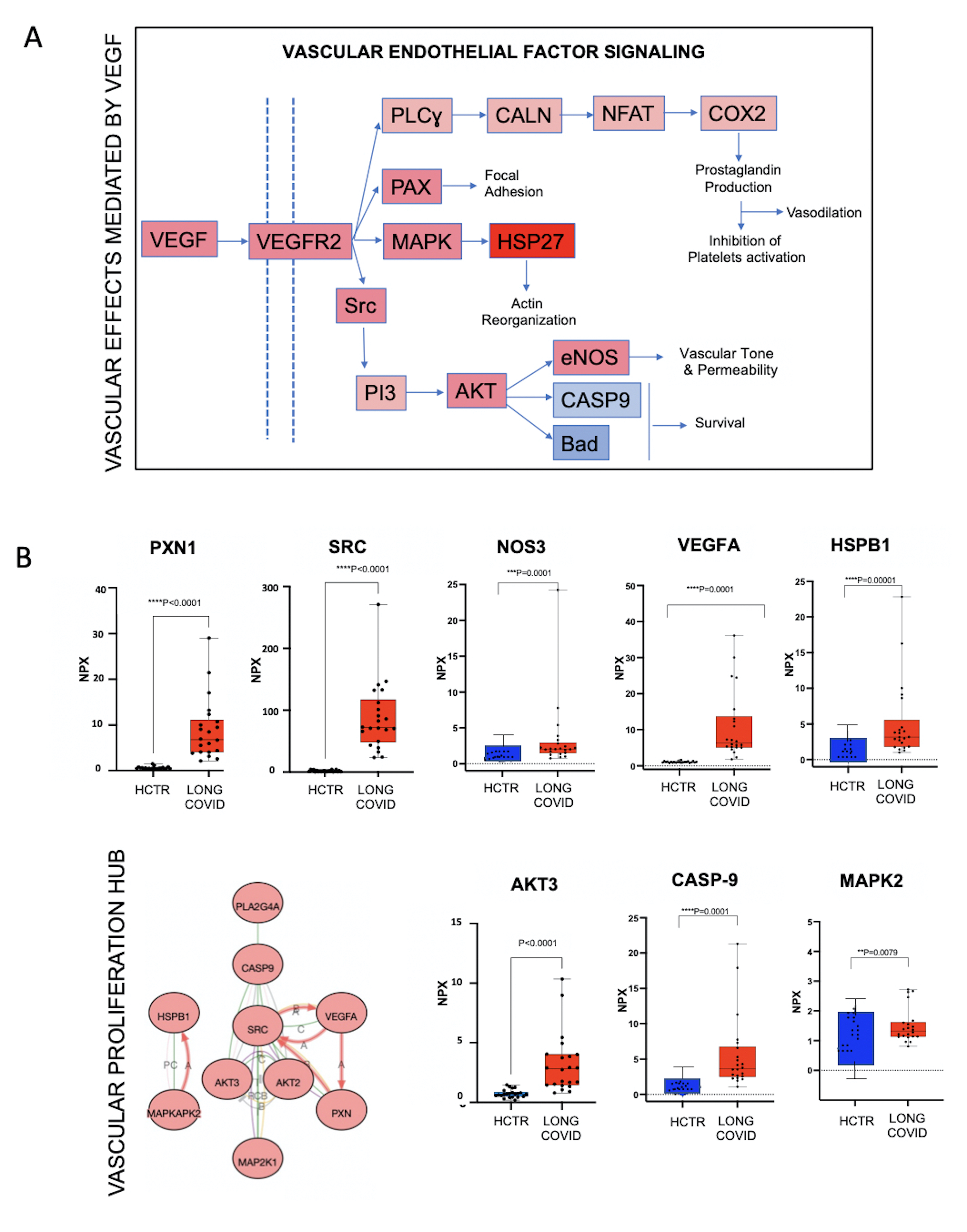

Supplement: Supplementary file 6 — Additional file 6: Figure S5. Resetting of cell type-associated protein abundance patterns in plasma may reflect vascular events mediated by VEGFA.VEGF signaling pathways as described by the KEGG are presented. Up-regulated biomarkers are in red and down-regulated biomarkers in blue. Analysis was done with KEGG Mapper and confirmed with iPathwayGuide software.Graphs show individual marker expression in Long-COVID plasma, as compared to healthy control subjects. Statistical significance was established using GraphPad-9, and P-value was considered significant if <0.05 in Mann-Whitney U test. Key markers were VEGFA, VEGFR2, AKT and MAPK, indicating endothelial cell activity, survival and migration. Key markers protein-protein interactions are presented in the diagram from the bottom panel, left. [file 12967_2023_4149_MOESM6_ESM.jpg]

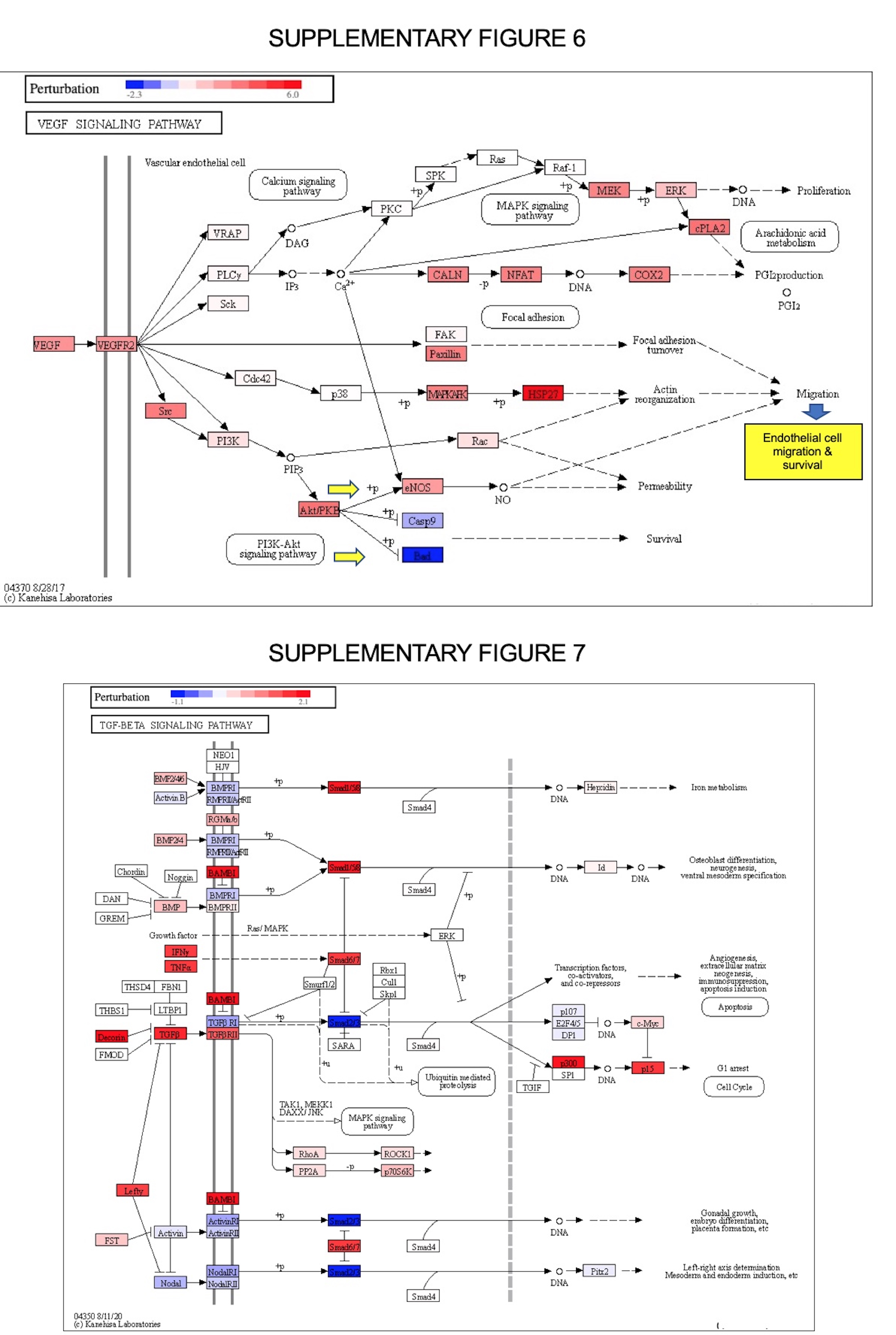

Supplement: Supplementary file 7 — Additional file 7: Figure S6. VEGF Signaling pathway. Figure S7. TGFB1 Signaling pathway. [file 12967_2023_4149_MOESM7_ESM.jpg]

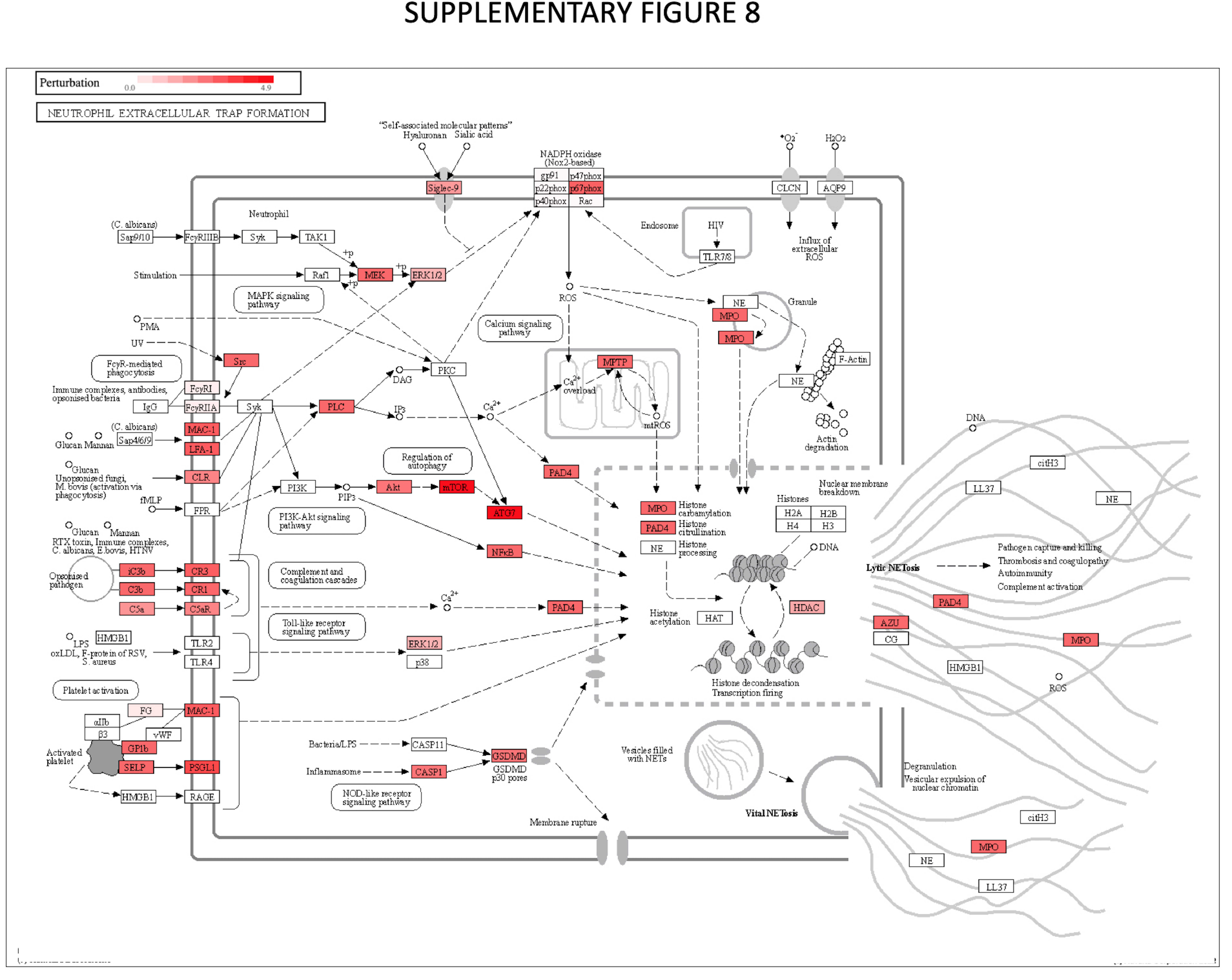

Supplement: Supplementary file 8 — Additional file 8: Figure S8. Neutrophil Extracellular Trap Formation. [file 12967_2023_4149_MOESM8_ESM.jpg]

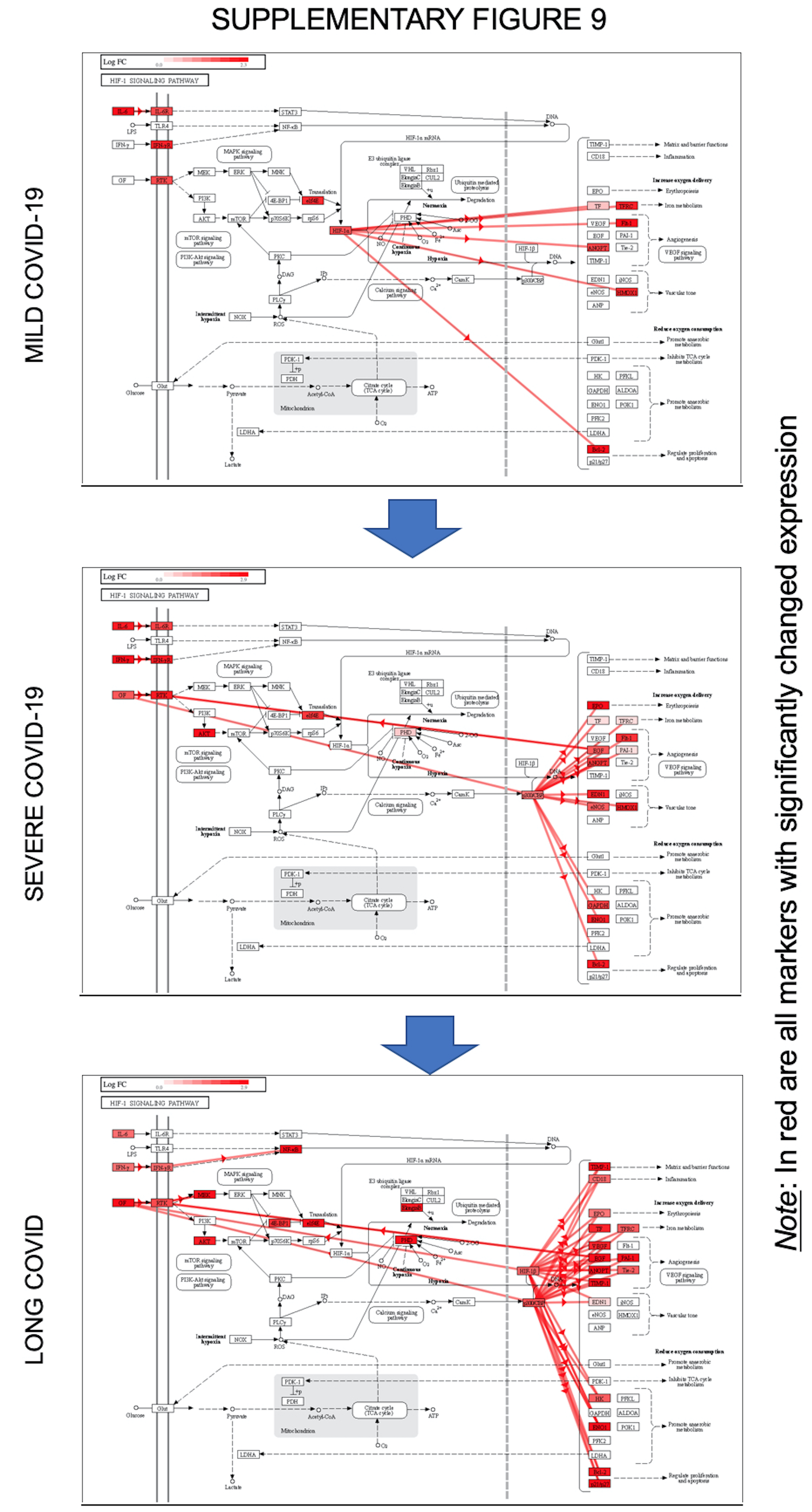

Supplement: Supplementary file 9 — Additional file 9: Figure S9. HIF1 Signaling pathways resulted from the pathways meta-analysis between HCTR, mild COVID-19, severe COVID-19 and Long-COVID groups. [file 12967_2023_4149_MOESM9_ESM.jpg]

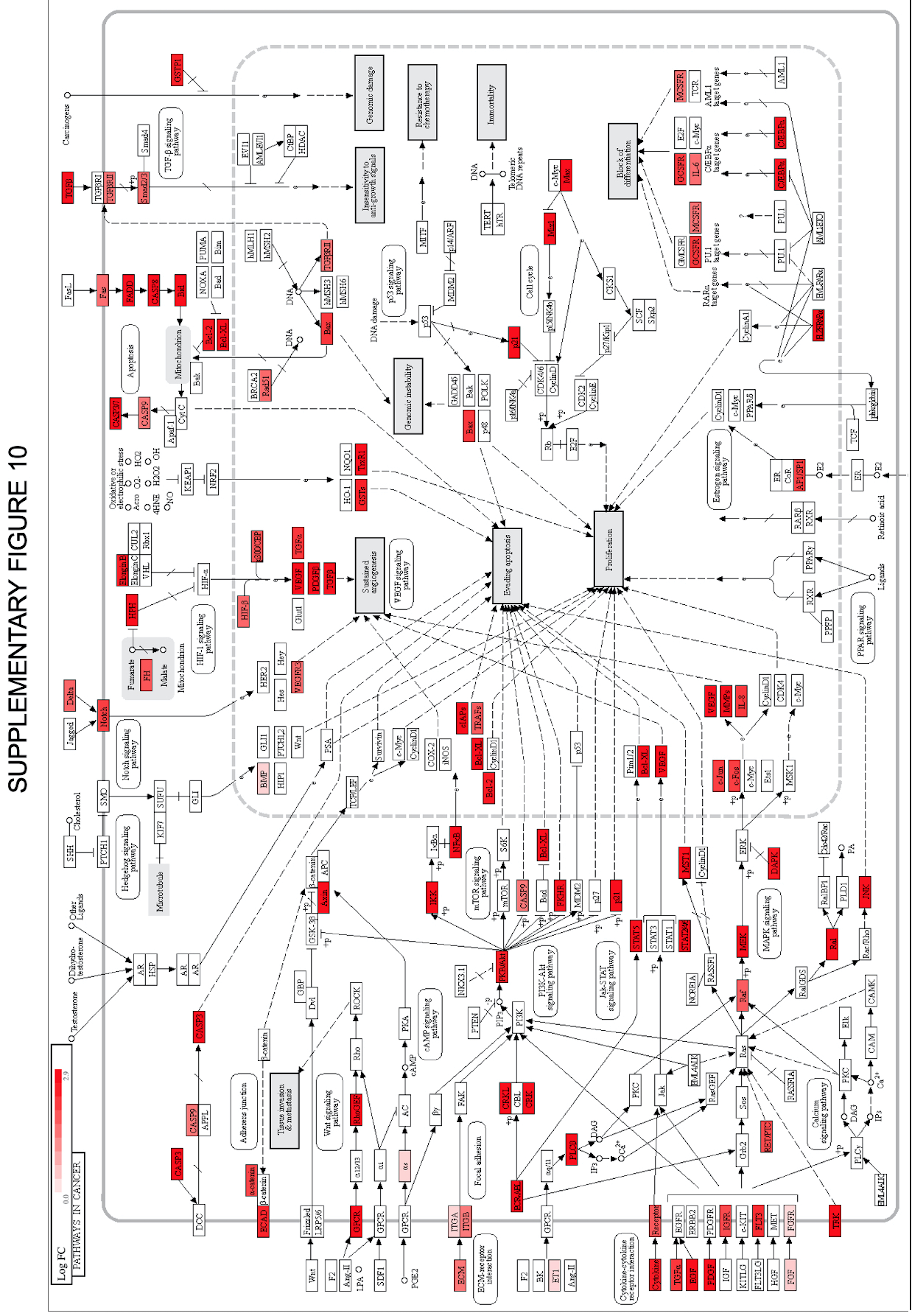

Supplement: Supplementary file 10 — Additional file 10: Figure S10. Cancer pathway and biomarkers that appear in Long-COVID patient plasma. [file 12967_2023_4149_MOESM10_ESM.jpg]

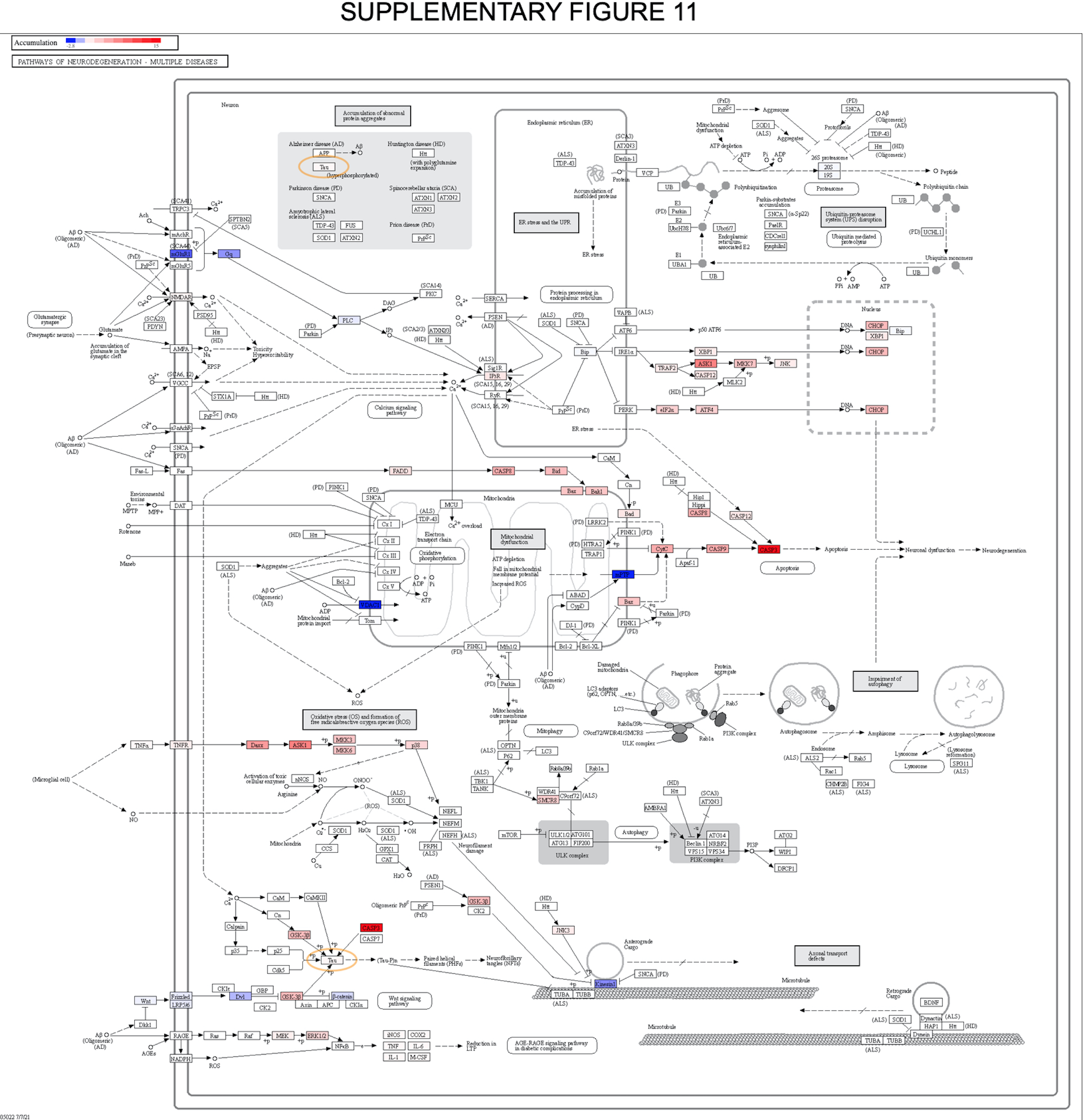

Supplement: Supplementary file 11 — Additional file 11: Figure S11. Neurological dysfunction pathway and biomarkers that appear in Long-COVID patient plasma. [file 12967_2023_4149_MOESM11_ESM.jpg]
